# Singlet Heterofission in Tetracene–Pentacene Thin‐Film Blends

**DOI:** 10.1002/anie.202007412

**Published:** 2020-09-30

**Authors:** Clemens Zeiser, Luca Moretti, Daniel Lepple, Giulio Cerullo, Margherita Maiuri, Katharina Broch

**Affiliations:** ^1^ Institute for Applied Physics University of Tübingen Auf der Morgenstelle 10 72076 Tübingen Germany; ^2^ Dipartimento di Fisica Politecnico di Milano Piazza Leonardo da Vinci 32 Milan Italy

**Keywords:** blends, energy transfer, singlet fission, thin films, time-resolved spectroscopy

## Abstract

Heterofission is a photophysical process of fundamental and applied interest whereby an excited singlet state is converted into two triplets on chemically distinct chromophores. The potential of this process lies in the tuning of both the optical band gap and the splitting between singlet and triplet energies. Herein, we report the time‐domain observation of heterofission in mixed thin films of the prototypical singlet fission chromophores pentacene and tetracene using excitation wavelengths above and below the tetracene band gap. We found a time constant of 26 ps for endothermic heterofission of a singlet exciton on pentacene in blends with low pentacene fractions, which was outcompeted by pentacene homofission for increasing pentacene concentrations. Direct excitation of tetracene lead to fast energy transfer to pentacene and subsequent singlet fission, which prevented homo‐ or heterofission of a singlet exciton on tetracene.

## Graphical abstract

A study of mixed thin films of pentacene and tetracene using ultrafast optical spectroscopy revealed spontaneous fission of a singlet exciton on pentacene, resulting in triplets on both chromophores, with a time constant of 26 ps. In blends containing pentacene fractions exceeding 5 %, the pentacene homofission process outcompetes heterofission because of the endothermicity of the latter.

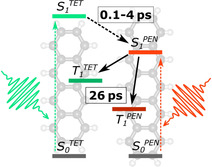

## Introduction

Singlet fission (SF) is the photophysical process whereby an excited singlet state is converted into two triplets on ultrafast timescales, ranging from femtoseconds to picoseconds,^[1]^ and has gained increasing attention in recent years due to its potential to overcome the Shockley‐Queisser limit of single junction solar cells.[^2^, ^3^, ^4^] Although the details of the SF mechanism are still debated, the role of intermolecular interactions in mediating the SF process has been emphasized.[^5^, ^6^, ^7^, ^8^]

For the efficient application of SF in solar cells, a balance between efficient generation of triplet pairs, facilitated by strong intermolecular interactions, and the subsequent separation of the triplet pairs into free triplets, for which weak intermolecular interactions are beneficial, has to be found.^[8]^ Different approaches are being followed for the development of new, promising SF compounds, often based on covalently bound dimers of SF chromophores, aiming at the optimization of the intermolecular coupling or the energy alignment of singlet and triplet states. These dimers or polymers of covalently bound SF chromophores exhibit intramolecular SF (iSF) and can be used to probe the impact of interaction strength and charge transfer state admixture on SF rates.[^9^, ^10^, ^11^, ^12^, ^13^, ^14^, ^15^]

Combining two different SF chromophores allows the engineering of the energy of singlet and triplet states with subsequent impact on SF[^12^, ^13^, ^14^] and can even lead to the observation of heterofission, which proceeds between the two SF subunits[^12^, ^13^, ^14^] and results in two triplet states localized on the different chromophores. Heterofission holds great potential for the application of SF in devices based on a controlled tuning of the exo‐ and endothermicity of the SF process and of the optical band gap.

**Figure 1:**
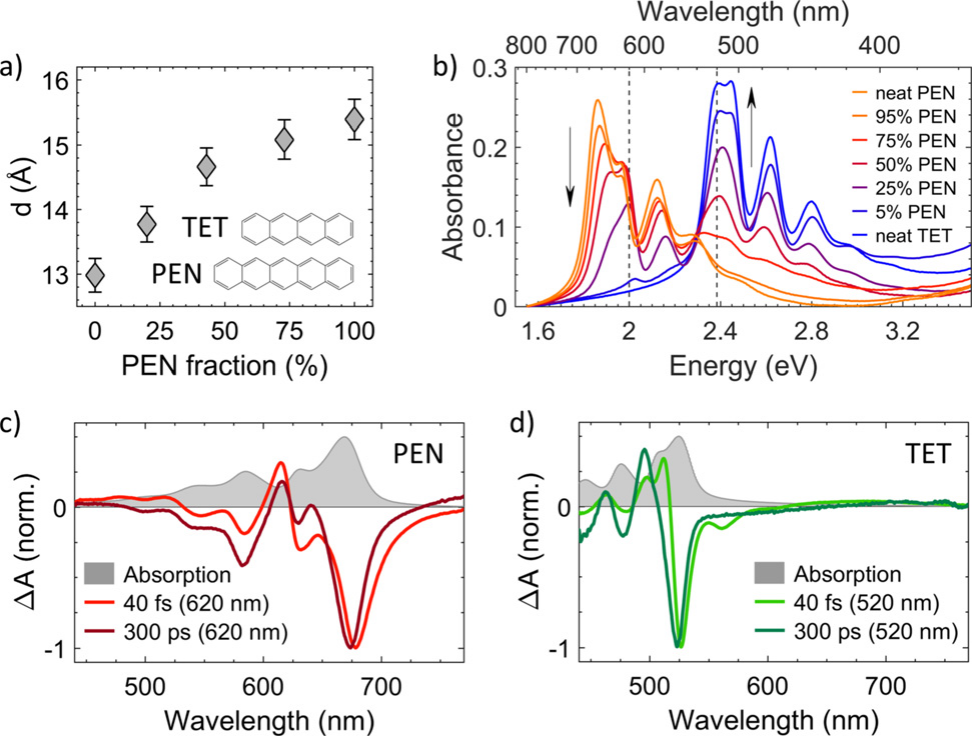
Structural and optical properties of blends of TET and PEN. a) Out‐of‐plane lattice spacing of the blends and chemical structure of TET and PEN. b) Absorption spectra of the blends. The two vertical, dotted lines show the excitation wavelengths used in the transient absorption (TA) spectroscopy measurements (620 nm and 520 nm). c,d) TA spectra of neat PEN (c) and neat TET (d) immediately after excitation (40 fs) and at long time delays (300 ps) with 620 nm (c) and 520 nm (d) excitation wavelength normalized to the maximum intensity. The corresponding absorption spectra are shown in light gray in the background.

The aim of our study is to continue this promising route by taking advantage of intermolecular heterofission in blends of SF chromophores.[^16^, ^17^, ^18^] We focus in particular on the prototypical SF chromophores pentacene (PEN) and tetracene (TET), whose structures are shown in Figure 1 a. These two materials have been shown early on to efficiently undergo intermolecular SF (xSF) in neat thin films and single crystals[^19^, ^20^] and iSF in dimers and polymers.[^9^, ^12^, ^13^, ^15^] While SF in TET is endothermic and occurs with a time constant of 80–90 ps,[^21^, ^22^] in PEN it is exothermic with an ultrafast time constant of 80 fs.[^23^, ^24^] By combining TET and PEN, a tuning of the exothermicity of SF has been demonstrated in covalently coupled oligoacenes.[^12^, ^13^] The possibility of intermolecular heterofission has been proposed in PEN‐doped TET single crystals already in the 1970’s[^16^, ^17^] but this process has never been observed in the time domain.

Here we investigate the photophysics of blends of TET and PEN prepared by organic molecular beam deposition, bridging the gap between the doping studies of the 1970’s[^16^, ^17^] and the recent studies based on iSF in oligoacene heterodimers.[^12^, ^13^] In particular, we aim to understand if the coherent SF pathway in PEN[^25^, ^26^, ^27^, ^28^, ^29^] still allows for heterofission in blends with PEN concentrations exceeding the doping level or if a similarly robust SF rate is observed as in blends of PEN with weakly interacting molecules.^[30]^ In blends with low (5 %) PEN fractions, we provide compelling evidence of a heterofission process which, due to its endothermic nature, occurs with a comparatively long (26 ps) time constant. In blends with higher PEN fractions, on the other hand, we observe that heterofission is outcompeted by PEN homofission.

## Results

Due to the similarity of the symmetry of the unit cell and the lattice parameters,[^31^, ^32^] it can be expected that upon co‐deposition TET and PEN form a solid solution characterized by the random occupation of lattice sites by molecules of either compound. This is supported by X‐ray reflectivity measurements which show a continuously increasing out‐of‐plane lattice spacing (Figure 1 a) with increasing PEN fraction. Steady‐state transmission spectroscopy on the blends (Figure 1 b) shows, for increasing PEN fractions, a decrease in the Davydov splitting of both compounds and changes in the relative intensities of the two Davydov components due to a reduced charge transfer state admixture.[^7^, ^33^]

We investigated the photophysical properties of the blends by transient absorption (TA) spectroscopy, using the results of the neat films (Figures 1 c,d) for comparison. In order to disentangle different photophysical processes in the blends, we used two excitation wavelengths: *λ*_exc_=620 nm, which is below the optical band gap of TET (Figure 1 b), and *λ*_exc_=520 nm, which excites both, PEN and TET.

### TA spectra of the neat compounds

The TA spectra of the neat films immediately after excitation (40 fs) and at long delays (300 ps) after the SF process has occurred are shown in Figures 1 c,d (for full TA maps see Figures S1, S2 and S16). For neat PEN upon excitation at *λ*_exc_=620 nm (Figure 1 c) the TA spectra are consistent with previous reports^[24]^ with the ground state bleach (GSB) of the electronically lowest excited state at 675 nm and the bleach of the high‐energy Davydov component and vibronic progression at shorter wavelengths. At short times (40 fs) we observe stimulated emission (SE) of singlets between 690 nm and 750 nm and singlet excited state absorption (ESA) below 500 nm. At longer times (300 ps) the singlet features disappear, and triplet ESA is observed above 700 nm.

For neat TET upon excitation at *λ*_exc_=520 nm (Figure 1 d and Figure S16) we observe the main GSB peak around 530 nm (40 fs after excitation) and 520 nm (300 ps after excitation) and, at early times, a contribution of SE between 550 nm and 570 nm, in agreement with literature.[^21^, ^22^] The rise in the GSB and the decay of the SE as well as the change in the shape and the position of the ESA above 700 nm are signatures of SF.[^21^, ^22^]

### TA spectra of TET:PEN blends upon excitation below the bandgap of TET

**Figure 2:**
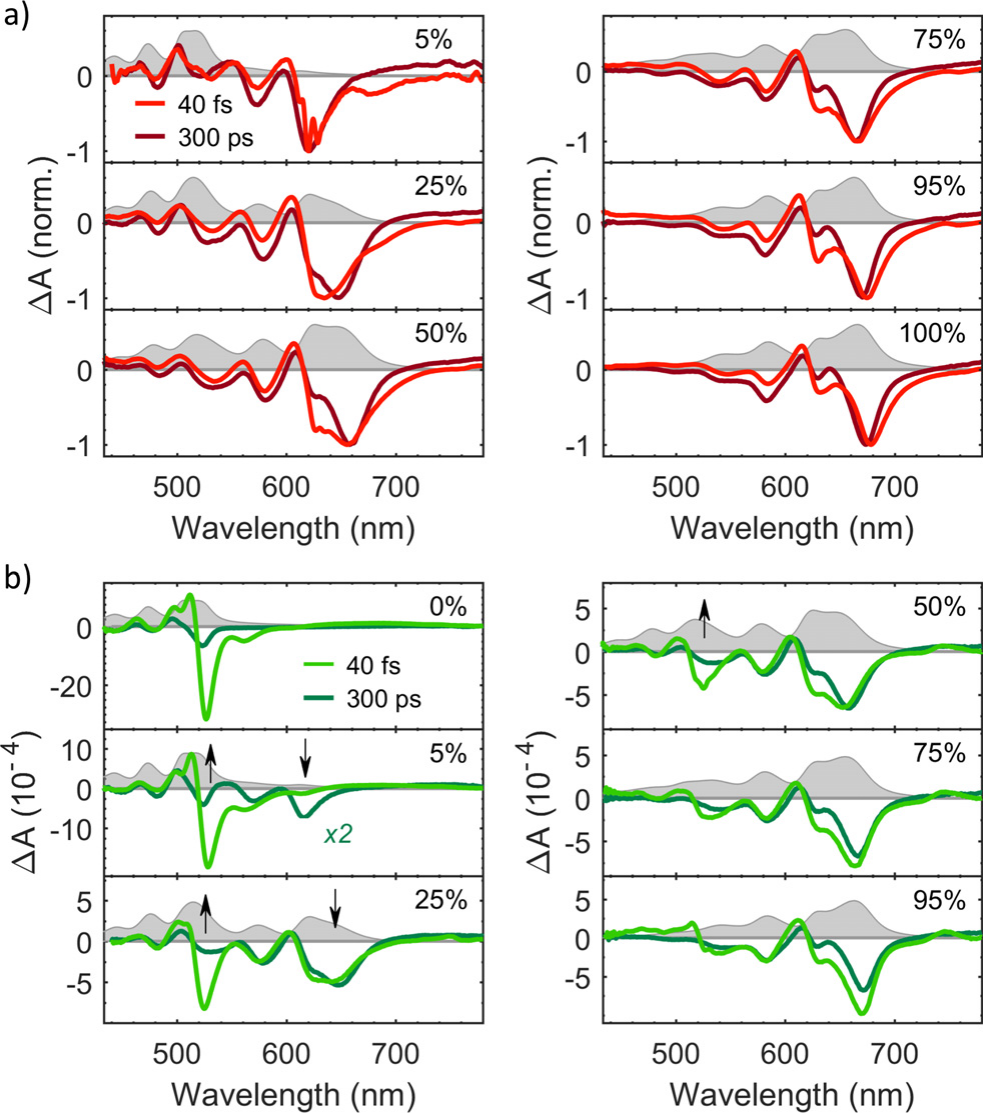
TA spectra of TET:PEN blends. a) Normalized TA spectra with *λ*_exc_=620 nm immediately after excitation (40 fs) and at long delays (300 ps). b) TA spectra with *λ*_exc_=520 nm immediately after excitation (40 fs) and at long delays (300 ps). Arrows indicate visible changes in the GSBs of PEN and TET. The corresponding absorption spectra are shown in light gray in the background. Note that the TA spectra in (a) are normalized for better comparison of the spectral shape but are not normalized in (b) to emphasize the effect of energy transfer.

In the first set of experiments (Figure 2 a), we used an excitation wavelength of *λ*_exc_=620 nm, which is below the optical band gap of TET. This allows us to exclude the possibility of energy transfer from excited TET to PEN and to probe only the relaxation pathways of PEN singlet excitons.

Compared to neat PEN, in the TA spectra of the blends the PEN GSB is shifted to shorter wavelengths with decreasing PEN concentration. The relative position and intensity of the two Davydov‐components of the PEN GSB at 675 nm and 630 nm change, until the energetically higher Davydov‐component dominates at 5 % PEN concentration. Furthermore, the intensity of the SE signal decreases with decreasing PEN fraction and its shape evolves from a shoulder at the low‐energy side of the GSB at high PEN fractions to a peak at 680 nm in the blend with 5 % PEN concentration. All these spectral changes are a direct result of the reduced interactions between neighboring PEN molecules and the reduced polarizability of the intermolecular environment with increasing TET concentration. Importantly, in the TA data of all blends at 300 ps we still observe triplet ESA at wavelengths above 700 nm, indicating the population of PEN triplet states through SF. Lastly, there are notable temporal evolutions of the spectral shape in the TA data of some blends. The shape of the GSBs of the 50 % PEN and the 25 % PEN blend differs between 40 fs and 300 ps (Figure 2 a). This change occurs within few tens of fs (Figure S1) and might originate from singlet exciton hopping to low energy sites as proposed for PEN:picene blends.^[30]^ Furthermore, the temporal evolution of the TA spectrum of the 5 % PEN blend in the range of the TET GSB at 515 nm is noteworthy. Comparing the spectrum at long delays (300 ps) with that immediately after excitation, a clear build‐up of a negative signal at the position of the TET GSB is observed. The implications of this observation will be discussed in more detail below.

**Figure 3:**
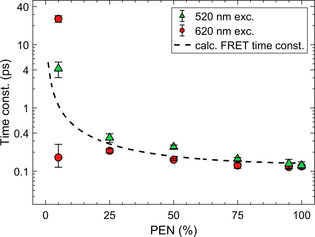
Time constants of the conversion of the initially excited singlet excitons to triplet excitons, obtained from GA of the measurements conducted with *λ*_exc_=520 nm (green) and *λ*_exc_=620 nm (red) excitation wavelength. The two data points shown for the 5 % PEN blend with *λ*_exc_=620 nm correspond to the two time constants obtained by GA, as discussed. FRET model (dashed line) accounting for energy transfer to nearest neighbors (Supporting Information).

We analyzed the TA‐data using a global analysis (GA)[^34^, ^35^] (Figures S7 and S9). Except for the 5 % PEN blend, this yields for all samples only two spectrally distinct species, which we assign to singlets and triplets. The time constants corresponding to the conversion of the excited singlets to the triplets are shown as red circles in Figure 3 and are assigned to SF (homo‐ or heterofission). For neat PEN, the GA gives a SF time constant of 119±5 fs, which is slightly higher than the SF time constant of 77±5 fs obtained by a fit of the single‐wavelength time trace at the wavelength of the PEN SE (Figure S4), the latter value being consistent with literature.^[24]^ Up to 25 % PEN concentration, the SF time constant in the blends increases slightly with decreasing PEN concentration to 210±17 fs. This trend changes dramatically in the blend with 5 % PEN concentration, where GA yields three distinct species, evolving into each other with time constants of 165 (−50/+100) fs and 26±3 ps, respectively. As will be discussed in more detail below, we have strong indications that the third, spectrally distinct species originates from heterofission, involving a singlet exciton on a PEN molecule and triplets on PEN and TET.

### TA spectra of TET:PEN blends upon excitation above the bandgap of TET

A second set of experiments (Figure 2 b) was performed using an excitation wavelength of *λ*_exc_=520 nm, above the TET band gap, which allows direct excitation of TET and energy transfer from TET to PEN. In addition to the PEN features, the TA spectra of blends with PEN fractions of 50 % and lower now show a strong TET GSB at 525 nm −529 nm at short times (40 fs) as expected for direct excitation of TET. The decay of this TET GSB is accompanied by a simultaneous rise of the PEN GSB in the blends with 25 % PEN or lower (arrows in Figure 2 b).

In order to determine the time constants of the dominating photophysical processes in the different blends, we performed a GA (Figures S8 and S10), which yielded two spectrally distinct species that can be assigned to singlets and triplets, as before. Comparing these results with the time constants extracted from the measurements with *λ*_exc_=620 nm (Figure 3), we find that the early photoinduced processes triggered at *λ*_exc_=520 nm occur with longer time constants. Importantly, the time constants obtained by GA for all blends except for the one with 5 % PEN are still more than two orders of magnitude smaller than TET homofission,[^21^, ^22^] which allows us to exclude TET homofission as a relevant relaxation channel in these blends.

Excitation with *λ*_exc_=520 nm directly populates the excited states of PEN and TET and, therefore, two different processes can occur simultaneously: first, SF from the PEN S_1_ and second, the transfer of excitation energy from TET to PEN via Förster resonance energy transfer (FRET) followed by SF. The time constants determined by GA reflect these two processes with different relative contributions.

As evident from the simultaneous decrease of the TET GSB and the increase of the PEN GSB and triplet ESA (for TA spectra and time traces with IR‐probe see Figures S12 and S13), the second relaxation channel (FRET + SF) significantly contributes to the photophysics in blends with high TET concentrations. We can further conclude that it outcompetes heterofission and TET homofission in blends with 25 % PEN concentration or higher, based on the observation that the shape of the TA spectra at long times is independent of the excitation wavelength (Figure S17). Taking the results obtained with *λ*_exc_=620 nm into account, the increase in time constants using *λ*_exc_=520 nm reflects predominantly the time constant of FRET, corresponding to the decay of the TET GSB and the rise of the PEN GSB.

In contrast, for neat PEN and blends with high PEN concentration, PEN homofission dominates the time constant due to a larger fraction of directly excited PEN molecules. The higher photon energy used causes the excitation of higher vibronic sublevels of S_1_ in PEN from which the exciton subsequently relaxes to the lowest vibrational level. Thus, the SF time constant is slightly longer compared to an excitation with *λ*_exc_=620 nm, see green triangles in Figure 3.

Lastly, the spectral features between 450 nm and 500 nm, which are observed in the TA spectra of the PEN:TET blends upon excitation with *λ*_exc_=620 nm and *λ*_exc_=520 nm, deserve some discussion as their position matches the vibronic progression of the GSB of neat TET. This might be surprising, as the excitation wavelength *λ*_exc_=620 nm lies well below the band gap of TET and we can also exclude two‐photon excitation of TET based on the results of a pump fluence dependence (Figures S14 and S15). However, for both PEN and TET an impact of local heating by the laser pulse on the shape of the TA spectrum has been reported,[^22^, ^24^, ^36^, ^37^] which can lead to long‐lived thermal artifacts and a TA spectrum that resembles the first derivative of the ground state absorption spectrum. To gauge the importance of this effect, we modeled the thermal artifact in the TA spectra of the 25 % PEN blend using the derivative of the steady‐state absorption spectrum[^36^, ^37^] (Figure S18). This is found to be fully sufficient to describe the spectral features for wavelengths below 500 nm. We therefore conclude that these features can be assigned to thermal artifacts and are not indicative of a delocalization of the singlet state.

### Evidence for singlet heterofission in the 5 % PEN blend

The probability of a given PEN molecule to have other PEN neighbors can be calculated assuming a random occupation of lattice sites by either molecule in the blends which follows a binomial distribution. In the 5 % PEN blend, 81 % of PEN molecules are fully isolated (0 PEN neighbors) and unable to undergo direct homofission, whereas 19 % have at least one PEN neighbor. As the dynamics of the 5 % blend differs significantly from those of the other blends, independent of the excitation wavelength, it was investigated in more detail.

**Figure 4:**
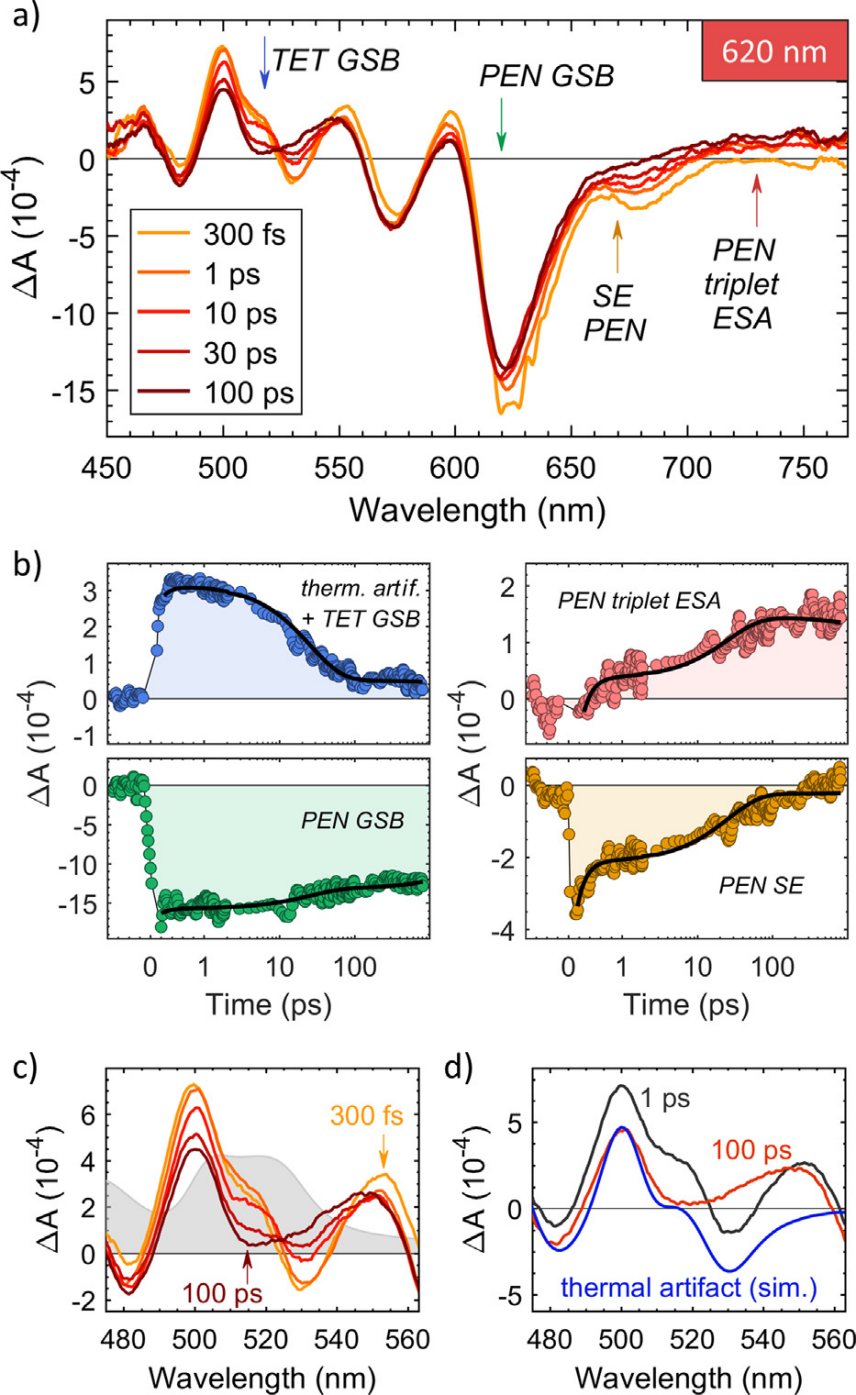
a) TA spectra and b) time traces of the blend with 5 % PEN using an excitation wavelength of *λ*_exc_=620 nm. The arrows in panel (a) indicate the wavelengths of extracted time traces in panel (b). The black lines in (b) are fit results of the GA. Data points affected by coherent artifacts have been removed in panel (b) for better visibility. Note the change from linear to logarithmic scale of the time axis at 1 ps. For residuals of the fits, see Figure S9. c) Evolution of the TA‐spectrum in the spectral region of the TET GSB. The absorption spectrum of the 5 % PEN blend in this region is shown in gray in the background. Note that the legend in (a) also applies to (c). d) Comparison of experimental TA‐data at two different delay times in the region of the TET GSB and the thermal artifact, simulated by the derivative of the steady‐state absorption spectrum.

We start the in‐depth analysis with the data with *λ*_exc_=620 nm (Figure 4), where we can exclude contributions from FRET and TET homofission. As mentioned before, GA yields three spectrally distinct species, which evolve into each other with two time‐constants (*τ*_1_=165 fs and *τ*_2_=26 ps). Based on the evolution associated spectra (EAS; Figure S7), the first, initially photoexcited species is assigned to singlet excitons on PEN, while the second and third species contain significant contributions from PEN triplet ESA and appear on time scales differing by two orders of magnitude. The single wavelength time trace extracted at the position of the PEN triplet ESA (730 nm) and the corresponding fit based on GA are shown in Figure 4 b. The bi‐exponential build‐up of the PEN triplet ESA with two time‐constants *τ*_1_ and *τ*_2,_ both of which are too fast for intersystem crossing,^[38]^ demonstrates the presence of two distinct SF channels. Comparing *τ*_1_ to the results of the analysis of blends with higher PEN concentrations (Figure 3) we assign it to homofission of the 19 % PEN molecules with a PEN neighbor.^[30]^ This leaves open the question of the origin of the second SF process connected to *τ*_2_. In order to shed light onto this pathway, in addition to the PEN triplet ESA we compared the fit results based on GA with time traces extracted at wavelength positions corresponding to the TET GSB at 514 nm, the PEN GSB at 619 nm and the PEN SE at 669 nm (Figure 4 b).

As can be seen in Figures 4 a,b, the signal from SE of isolated PEN molecules remains after the first SF process has been completed, but it is absent at delay times >300 ps, indicating that the second relaxation pathway involves the decay of singlets on isolated PEN molecules. This could be caused by the following processes: (i) radiative relaxation of the PEN singlet states (ii) energy transfer from PEN monomers to PEN dimers, and (iii) heterofission.[^16^, ^17^]

We can exclude radiative relaxation since the PEN SE decay is not accompanied by a corresponding decrease in the PEN GSB. This indicates the depopulation of the PEN excited state towards a long‐lived state which is not the PEN ground state. Furthermore, the time scales of the bi‐exponential decrease of the PEN SE are almost two orders of magnitude faster than the radiative relaxation of monomeric PEN in a weakly interacting picene matrix (*τ*=1.17 ns^[39]^). Energy transfer to PEN dimers would lead to a red shift of the PEN GSB, which is not observed in our data (Figure 4 a).

This leaves heterofission between an excited PEN monomer and its TET neighbor, creating triplets on PEN and TET, as the most likely decay channel for the PEN singlets.

This scenario is consistent with the rise of the PEN triplet ESA simultaneously to the decay of the PEN SE but without affecting the PEN GSB. The long‐lived state which is populated is then the triplet state of PEN. The temporal evolution of the TA spectra in the region of the TET GSB (500–550 nm) further supports this interpretation, as we observe a clear build‐up of a negative signal within *τ*_2_ along with an isosbestic point^[40]^ at 525 nm (Figures 4 c,d). Again, this indicates a bleach of TET molecules due to the formation of a long‐lived state, such as a triplet. Since PEN homofission does not involve TET and occurs with the time scale *τ*_1_ on which there is almost no change in the signal at 514 nm, it cannot explain the observed build‐up of the TET GSB and the thermal artifact, although pronounced in this wavelength region, can be well separated from the TET GSB at long times (Figures S18 and S19).

**Figure 5:**
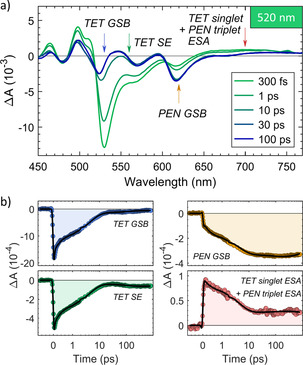
TA spectra of the blend with 5 % PEN using an excitation wavelength of *λ*_exc_=520 nm. The arrows in panel (a) indicate the wavelengths of extracted time traces in panel (b). The black lines in (b) are fit results of the GA. Note the change from linear to logarithmic scale of the time axis at 1 ps. For residuals of the fits, see Figure S10.

In the TA data measured with *λ*_exc_=520 nm of the blend with 5 % PEN (Figure 5) we observe complex multi‐exponential dynamics that reflect the relaxation of excited PEN and TET molecules via a multitude of channels (Figure S8). The simultaneous decrease in the TET GSB and rise in the PEN GSB again points towards the transfer of excitation energy via FRET and the build‐up of triplet ESA indicates the population of the triplet state of PEN by SF. This can occur via PEN homofission from directly excited PEN dimers, via FRET to PEN dimers followed by PEN homofission or FRET to isolated PEN molecules and subsequent heterofission. The persistence of the TET GSB at long delays is again evidence for a long‐lived state, such as a TET triplet, which can be populated either via heterofission or TET homofission.

One clear indication for heterofission in the 5 % PEN blend with *λ*_exc_=620 nm was the build‐up of a TET GSB, which is at *λ*_exc_=520 nm counteracted by FRET that leads to a decrease in the TET GSB intensity. This prevents a clear disentanglement of these concomitant processes in the experiment with *λ*_exc_=520 nm.

## Discussion

**Figure 6:**
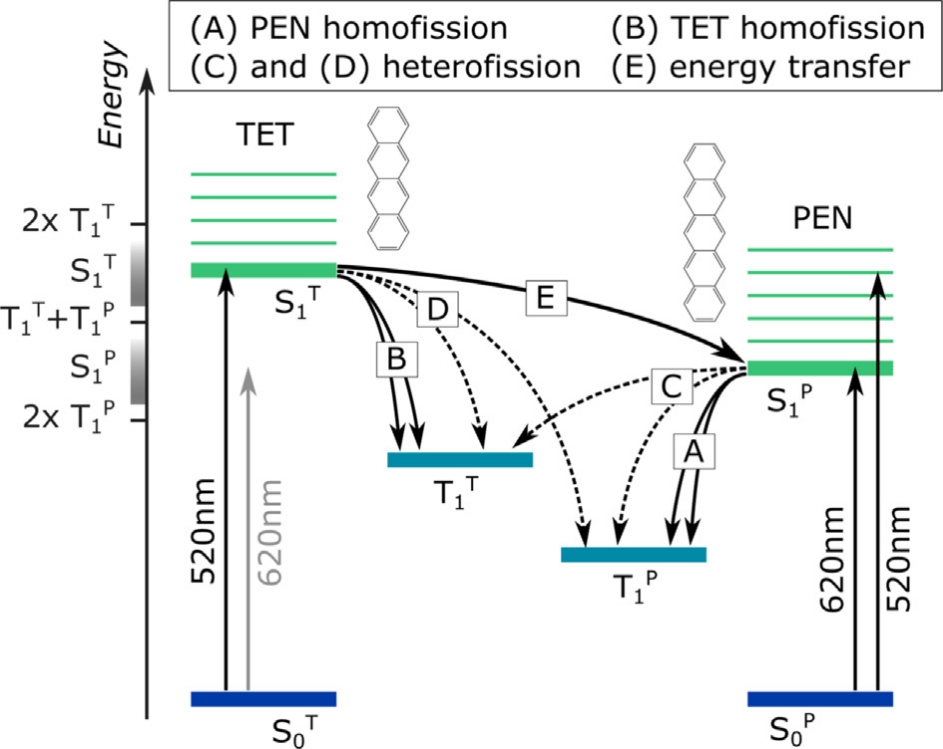
Possible ultrafast (sub‐ns) processes involving excited states of TET and PEN.

The possible ultrafast (sub‐ns) relaxation pathways in TET:PEN blends and the respective time constants are summarized in Figure 6 and Table 1. As discussed before, we have clear evidence for heterofission in the blend with 5 % PEN and *λ*_exc_=620 nm on a timescale of *τ*_C_=26 ps or a rate of *r*_C_=0.038 ps^−1^. The reduced rate of heterofission compared to PEN homofission can be explained by the endothermicity of the process given by Δ*E*=*E*(T_1_^PEN^)+*E*(T_1_^TET^)−*E*(S_1_^PEN^). *E*(S_1_^PEN^)=2.02 eV (Figure 1 b) is determined from the maximum of the PEN absorption in the 5 % blend. The blue shift of *E*(S_1_^PEN^) by 180 meV compared to PEN in neat thin films arises from a change in the polarizability of the molecular environment, which does not significantly affect the energy of the triplet states *E*(T_1_).^[41]^ Thus, *E*(T_1_^PEN^) and *E*(T_1_^TET^) can be taken from literature.[^23^, ^42^] This gives an endothermicity of the heterofission process of Δ*E*=90 meV and also explains why it is faster than TET homofission (Δ*E*=180 meV).^[42]^ The difference in timescales compared with intramolecular heterofission (*τ*_C_=1 ps) observed in oligoacenes[^12^, ^13^] can be explained by the weaker coupling in the mixed thin films.

**Table 1:** Time constants of the photophysical processes observed in the blends (labeled as in Figure 6)

	*λ*_exc_=620 nm		*λ*_exc_=520 nm	
	5 % PEN	>5 % PEN	5 % PEN	>5 % PEN
*τ*_A_	165 fs*	110–210 fs	m.e.	120–340 fs
*τ*_E_	n.a.	n.a.		
*τ*_B_	n.a.	n.a.		X
*τ*_C_	26 ps	X		X
*τ*_D_	n.a.	n.a.	X	X

Key: processes that cannot be observed due to the excitation wavelength (n.a.), processes that cannot be disentangled due to the multi‐exponential convoluted nature of the signal with excitation at 520 nm (m.e.; Supporting Information), and processes that are outcompeted by others (X). *This time constant has a large uncertainty (−50/+100 fs).

For blends with PEN concentrations exceeding 5 % and *λ*_exc_=620 nm we find no evidence for heterofission. Based on the endothermicity of the heterofission process compared to the exothermic PEN homofission, it is not surprising that the latter outcompetes the first in blends with a high probability of PEN molecules with one or more PEN neighbors. The time constants we derive from GA are within the range of previous reports of PEN homofission in PEN:spacer blends^[30]^ and, thus, there is no indication for an impact of the presence of TET molecules on PEN SF in these blends. It is noteworthy that PEN homofission seems to be in general robust against the incorporation of weakly interacting compounds and independent of the molecular geometry, the orientation of the transition dipole moment or the optical band gap. This is most likely a direct consequence of the fact that SF in PEN proceeds via a coherent pathway,[^28^, ^29^] where the triplet pair state directly mixes into the photoexcited bright state. This direct mixing of states involving two PEN molecules is a further rationale for the lack of heterofission in TET:PEN blends with PEN concentrations exceeding 5 %.

The interpretation of the TA data obtained with an excitation wavelength of *λ*_exc_=520 nm becomes complex as TET homofission and FRET from TET to PEN additionally contribute to the dynamics. For blends with a higher PEN concentration, FRET followed by PEN homofission dominates the dynamics due to a large density of PEN acceptors. This channel outcompetes TET homofission and also heterofission from an excited TET molecule, as seen by the lack of TET GSB and the overall similarity of the TA spectra with *λ*_exc_=520 nm and *λ*_exc_=620 nm at long times (Figure S17). This is surprising, as heterofission from TET is exothermic by 200 meV, but underlines the importance of additional parameters such as orbital overlap and coupling between the singlet and the triplet‐pair state. FRET and PEN homofission occur on similar time scales which thus cannot be separated, and we obtain an overall time constant ranging from 120–340 fs for the different mixing ratios for the decrease of the TET GSB and the rise of the PEN triplet ESA.

## Conclusion

To conclude, we investigated the photophysics of mixed thin films of TET and PEN using TA spectroscopy, selectively pumping either PEN alone or PEN and TET simultaneously. As proposed before for PEN‐doped TET single crystals,[^16^, ^17^] we observed heterofission of a singlet exciton on a PEN molecule to two triplets on one PEN and one TET molecule, after direct excitation of PEN (*λ*_exc_=620 nm). This phenomenon occurs in blends with PEN concentrations of 5 % with a comparatively slow time constant of *τ*=26 ps due to its endothermicity of 90 meV. On the other hand, in blends with higher PEN concentrations, the coherent pathway of PEN homofission prevents heterofission and results in time constants of PEN SF similar to those observed for other PEN blends.[^30^, ^43^] Lastly, excitation at *λ*_exc_=520 nm of blends with a PEN concentration of 25 % or higher leads to PEN homofission (for directly excited PEN molecules) and FRET from TET to PEN followed by PEN homofission (for excited TET molecules) on timescales of 120–340 fs. In the 5 % PEN blend different possible processes coexist on time scales on the order of 4.2 ps, which cannot be disentangled: PEN and TET homofission, FRET from TET to PEN and subsequent SF (PEN homo‐ or heterofission). In all blends, FRET prevents heterofission of a TET singlet exciton.

Besides the determination of heterofission time constants in mixed thin films of small molecules, we were able to show that heterofission is observable in weakly interacting systems going beyond studies of doped single crystals[^16^, ^17^] or strongly coupled systems such as heterodimers.[^12^, ^13^] Nevertheless, we also demonstrated that coherent, exothermic homofission can outcompete heterofission if it becomes an accessible relaxation pathway. As a perspective for future applications, our approach bridges the gap between oligoacenes and single crystals. It allows a continuous tuning of the optical band gap along with the singlet and triplet energies by varying the mixing ratio without significant detrimental impact on homofission time constants.

## Conflict of interest

The authors declare no conflict of interest.

## Supporting information

As a service to our authors and readers, this journal provides supporting information supplied by the authors. Such materials are peer reviewed and may be re‐organized for online delivery, but are not copy‐edited or typeset. Technical support issues arising from supporting information (other than missing files) should be addressed to the authors.

Supplementary
