# Supplementary material for: Singlet Heterofission in Tetracene–Pentacene Thin‐Film Blends
Source: Angew Chem Int Ed Engl. 2020 Sep 30;59(45):19966–73. doi: 10.1002/anie.202007412 (PMC7820980; doi:10.1002/anie.202007412)
Supplement: Supplementary file 1 — Supplementary [file ANIE-59-19966-s001.pdf]

Angewandte Chemie
Eine Zeitschrift der Gesellschaft Deutscher Chemiker

Supporting Information

Singlet Heterofission in Tetracene–Pentacene Thin-Film Blends

Clemens Zeiser[+], Luca Moretti[+], Daniel Lepple, Giulio Cerullo, Margherita Maiuri, and Katharina Broch**

anie_202007412_sm_miscellaneous_information.pdf

Table of Contents

Experimental Procedures 2
TA maps (620 nm excitation) 3
TA maps (520 nm excitation) 4
Time traces (620 nm excitation) 5
Time traces (520 nm excitation) 7
Global analysis (620 nm excitation) 9
Global analysis (520 nm excitation) 10
Comparison of fits and time traces of the 5% PEN blend and fit residuals 11
Time trace fits with non-exponential triplet decay 13
IR-probe TA measurements (520 nm excitation) 15
Comparison of 25% PEN blends (mixed with TET and PIC) and variable pump energy 16
TA measurements on neat TET 17
Comparison of 300 ps spectra of TA measurements with 620 nm and 520 nm excitation 18
Thermal artifacts 19
Quantiative calculation of spectral contributions in the 5% PEN blend (λ_{exc} = 620nm) 20
Förster radius 21
Förster resonance energy transfer rate 21
References 22

Experimental Procedures

The mixed films of TET (Sigma Aldrich, 99.9%) and PEN (Sigma Aldrich, 99.99%, triple sublimed) were grown by organic molecular beam deposition on silicon with a native oxide layer and on borofloat glass substrates at a base pressure of 1×10^{-9} mbar. The total growth rate was 0.6 nm/min, with the rates of the two materials monitored separately by two quartz crystal microbalances, calibrated using X-ray reflectivity. The final film thickness was 80nm.
X-ray reflectivity was measured on a diffractometer (3303TT, GE) using Cu Kα-radiation (λ = 1.5406 Å) and a 1D detector (Meteor 1D, XRD Eigenmann). UV-vis transmission spectra were measured using a Perkin Elmer Lambda 950 spectrophotometer.
Ultrafast pump probe experiments were performed using a Ti:Sapphire chirped pulse amplified source (Coherent Libra), with 4mJ output energy, 1 kHz repetition rate, 800 nm central wavelength and 100 fs pulse duration. Excitation pulses at 620 nm with 70-fs duration were generated by non-collinear optical parametric amplification in a β-barium borate crystal. Pump pulses were focused to a 350 μm diameter spot. Probing was achieved in the visible region (430 nm – 780 nm) by using a white light continuum generated in a thin sapphire plate. Transient transmission spectra were collected by using a fast optical multichannel analyzer. The measured quantity is the differential transmission, ΔT/T, from which the differential absorbance is calculated as: $\Delta A \cong -\Delta T/T$. Excitation energy has been kept for λ_{exc} = 620 nm at 70 nJ, for λ_{exc} = 520 nm at 20 nJ, corresponding to fluences of 73 $\mu J/cm^2$ and 21 $\mu J/cm^2$, respectively. Measurements were performed at room temperature and under vacuum to avoid sample degradation. For the global analysis of the obtained TA data the open source software Glotaran was used.[1]

TA maps (620 nm excitation)

Full transient absorption (TA) maps of pentacene (PEN):tetracene (TET) blends and neat PEN with an excitation wavelength of 620 nm are shown in Figure S1.

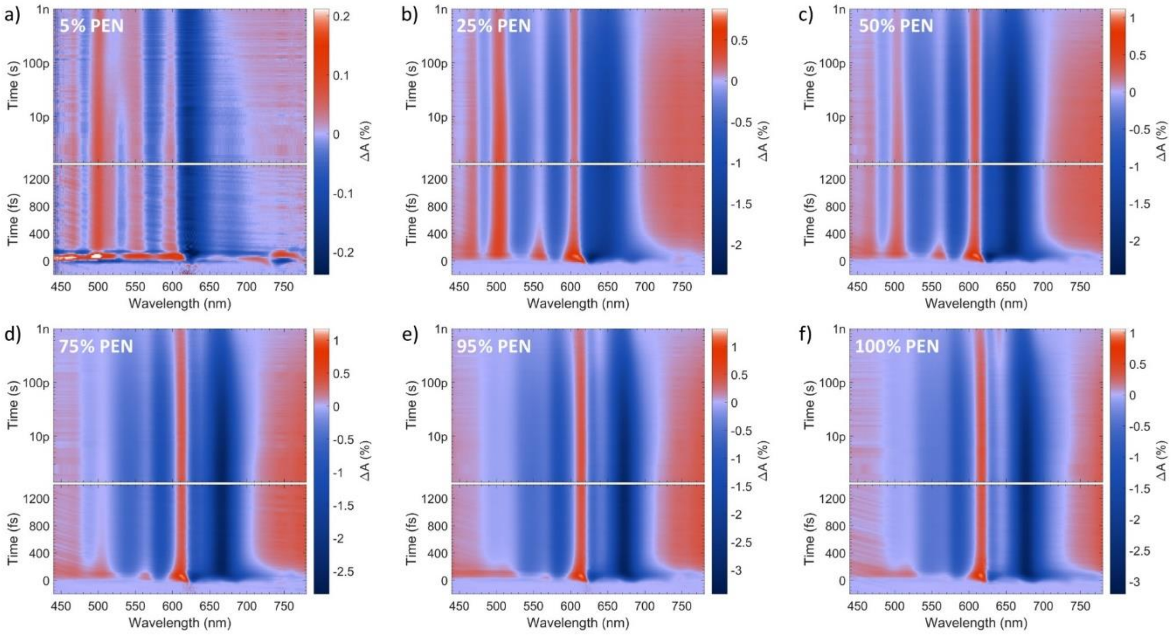

Figure S1: Time-wavelength maps of TA measurements of TET:PEN blends with 620 nm excitation wavelength, 70 nJ pump pulse energy and different PEN concentrations.

TA maps (520 nm excitation)

Full TA maps of PEN:TET blends and neat PEN with an excitation wavelength of 520 nm are shown in Figure S2.

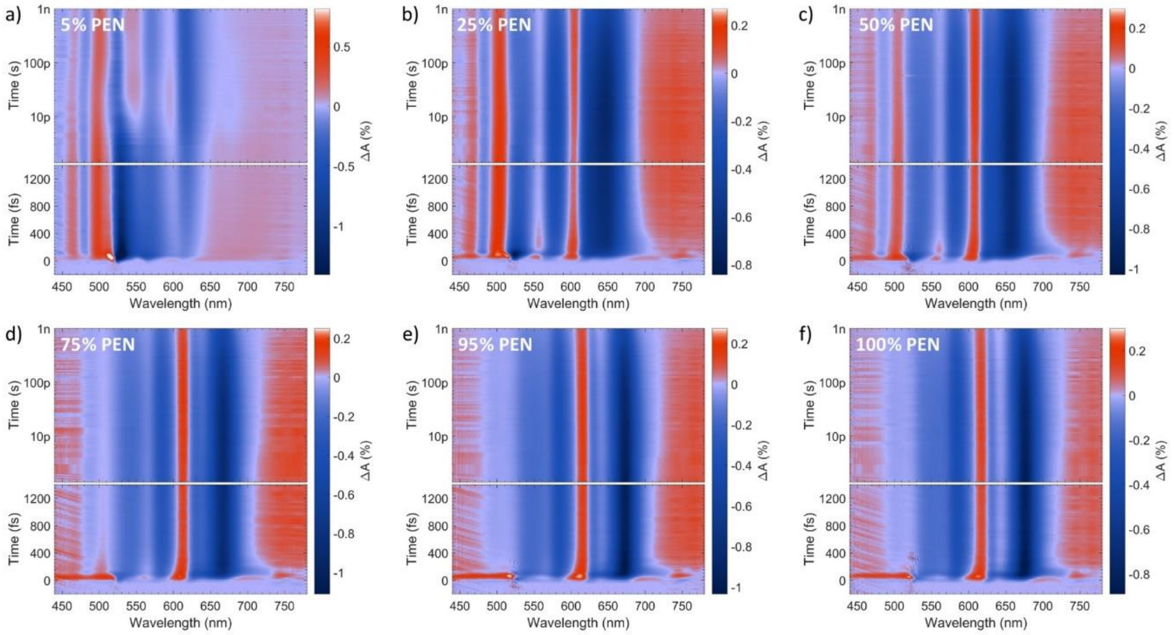

Figure S2: Time-wavelength maps of TA measurements of TET:PEN blends with 520 nm excitation wavelength, 20 nJ pump pulse energy and different PEN concentration.

Time traces (620 nm excitation)

Time traces extracted from TA maps (Figure S1) measured using an excitation wavelength of 620 nm are shown in Figure S3. The chosen wavelengths represent the build-up of the triplet excited state absorption (ESA) at λ=730 nm (5% PEN) and λ=780 nm and λ = 587 nm (others), the region of the PEN ground state bleach (GSB) and stimulated emission (SE) at λ = 595 – 678 nm and singlet ESA of PEN and TET GSB at λ = 482 nm – 514 nm. Especially in the triplet ESA and the PEN GSB region, the slower singlet fission (SF) rate of PEN in blends with lower PEN concentration is well visible. Please note that the short wavelength regions (λ = 538 nm and λ = 514 nm) were chosen, since they allow to see the dynamics of the TET contribution in the 5% blend.

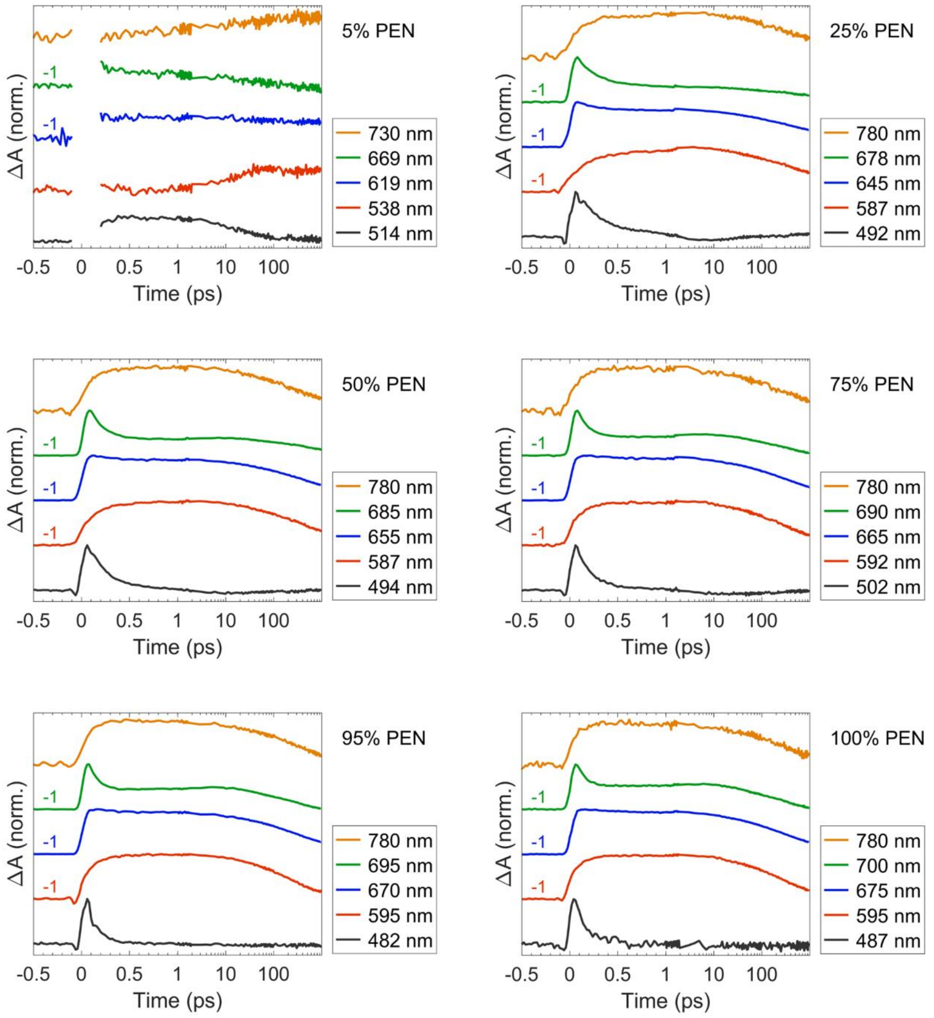

Figure S3: Dynamics at different wavelengths of TA measurements of different TET:PEN blends excited at 620 nm. Traces with a negative ΔA signal are shown positive and are labeled with -1. Due to a large coherent artifact the time traces of the 5% PEN blend are only shown after 200 fs.

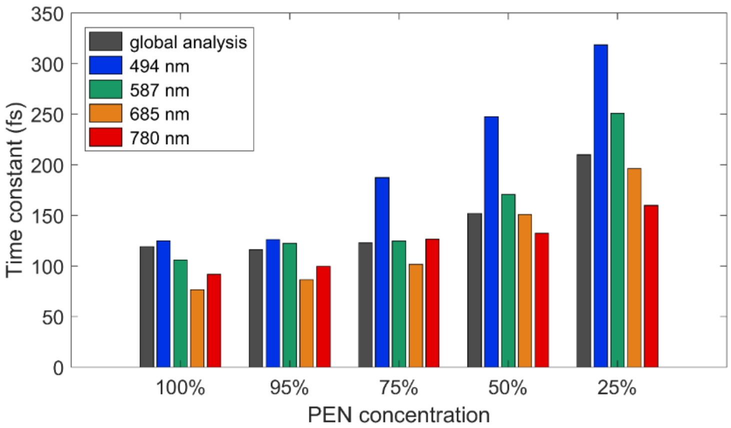

Figure S4: Time constants obtained from exponential fits of time traces extracted at different wavelengths from the TA-maps (Figure S1). The wavelengths reported in the legend correspond to the wavelength of the time traces of 75 % PEN blends. The wavelengths of the time traces of the other blends are chosen according to the spectral shift.

Time traces (520 nm excitation)

Time traces extracted from TA maps (Figure S2) measured using an excitation wavelength of 520 nm are shown in **Figure S3**Figure S5. The dynamics at λ = 700 nm (5% PEN) and λ = 770 nm and λ = 530 nm (others) contain contributions from both the TET singlet and the PEN triplet ESA and it is therefore difficult to identify energy transfer dynamics. The PEN GSB (620 – 675 nm), however, shows a slower build-up in blends with lower PEN concentration, which corresponds to the increasing population of PEN excitons. In the range of the TET GSB (530 nm), we observe fast dynamics in all blends, originating from excited PEN excitons undergoing homofission. Besides the fast dynamics, there are slower decays in the 5% and the 25% blend originating from energy transfer. Fits of selected time traces are shown in Figure S6.

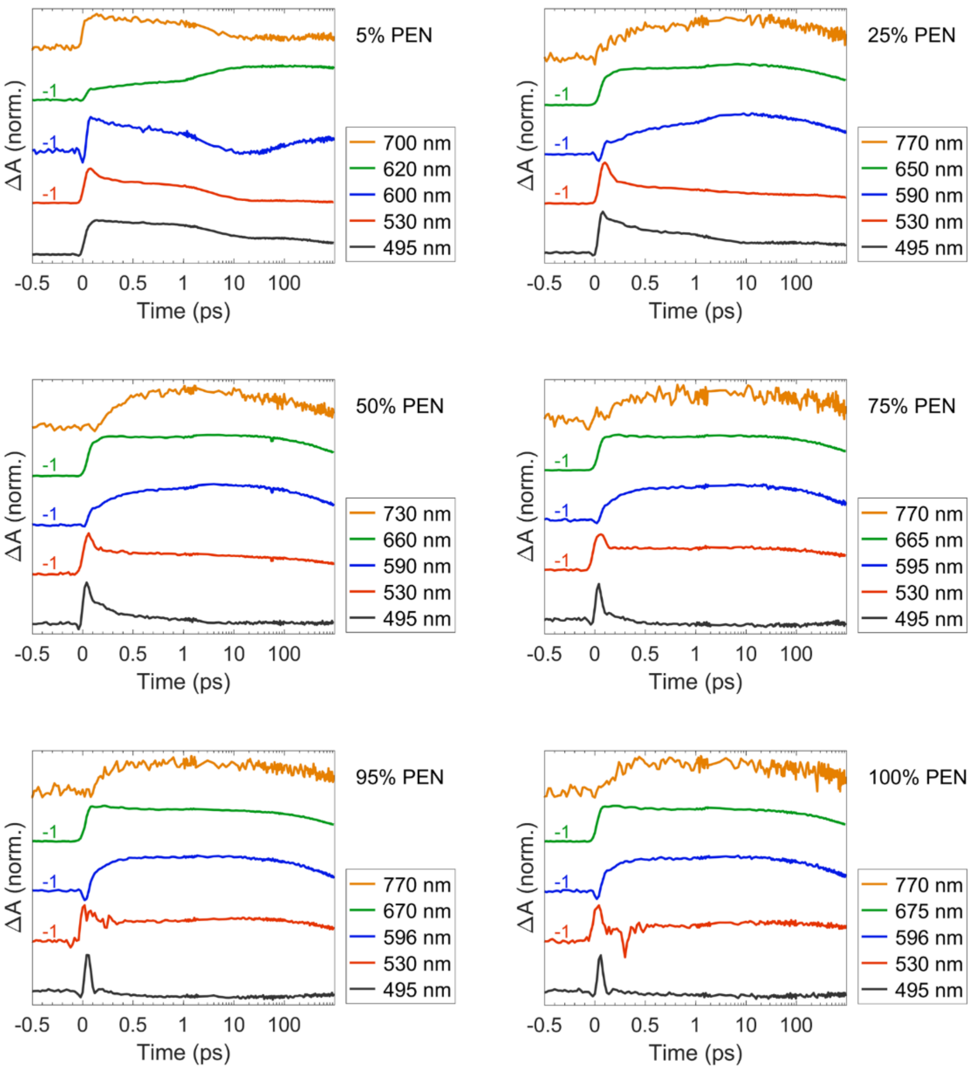

Figure S5: Dynamics at different wavelengths of TA measurements of different TET:PEN blends excited at 520 nm. Traces with a negative ΔA signal are shown positive and are labeled with -1.

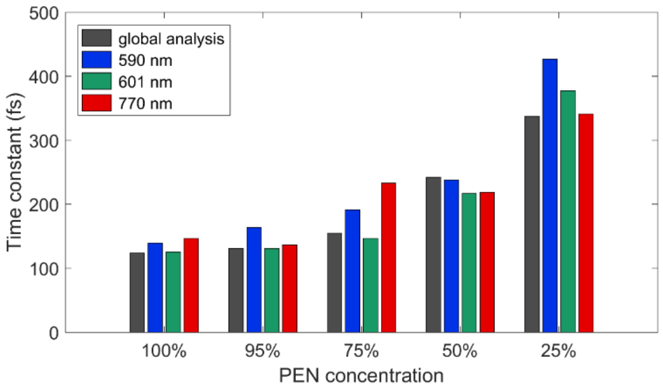

Figure S6: Time constants obtained from exponential fits of time traces extracted at different wavelengths from the TA-maps (Figure S2). The wavelengths reported in the legend correspond to the wavelength of the time traces of 75 % PEN blends. The wavelengths of the time traces of the other blends are chosen according to the spectral shift.

Global analysis (620 nm excitation)

The results of the global analysis (GA) of the TA measurements of PEN:TET blends and neat PEN with 620 nm excitation wavelength are shown in Figure S7. For a good fit of the whole measurement until 1 ns, four evolution associated spectra (EAS) are necessary for the blends with PEN concentration of 25% or more. The last three EAS are, however, very similar in shape, which is consistent with the non-exponential decay of triplets reported in Refs. 2-4, and only the first and second EAS differ significantly for each of these blends. For the 5% PEN blend, the whole TA map could instead be fitted using only three EAS, all of which are distinct from each other. We therefore conclude that only two distinct species are involved in the photophysics of blends with 25% PEN concentration or more: 1) PEN singlets with a SF time constant of τ_1. 2) PEN triplets with a non-exponential decay modelled by three exponential decays (τ_2, τ_3, τ_4). Further discussion of the triplet decay and its dependence on the mixing ratio is beyond the scope of this study. In the analysis of the blend with 5% PEN we identify three EAS resulting from different combinations of species: 1) Singlet excitons on isolated PEN molecules and PEN dimers. 2) Singlet excitons on isolated PEN molecules and triplets on PEN dimers formed by PEN SF with τ_1. 3) Triplets formed by PEN homofission and in addition triplets on both PEN and TET formed by heterofission with τ_2. Both types of triplets decay with τ_3.

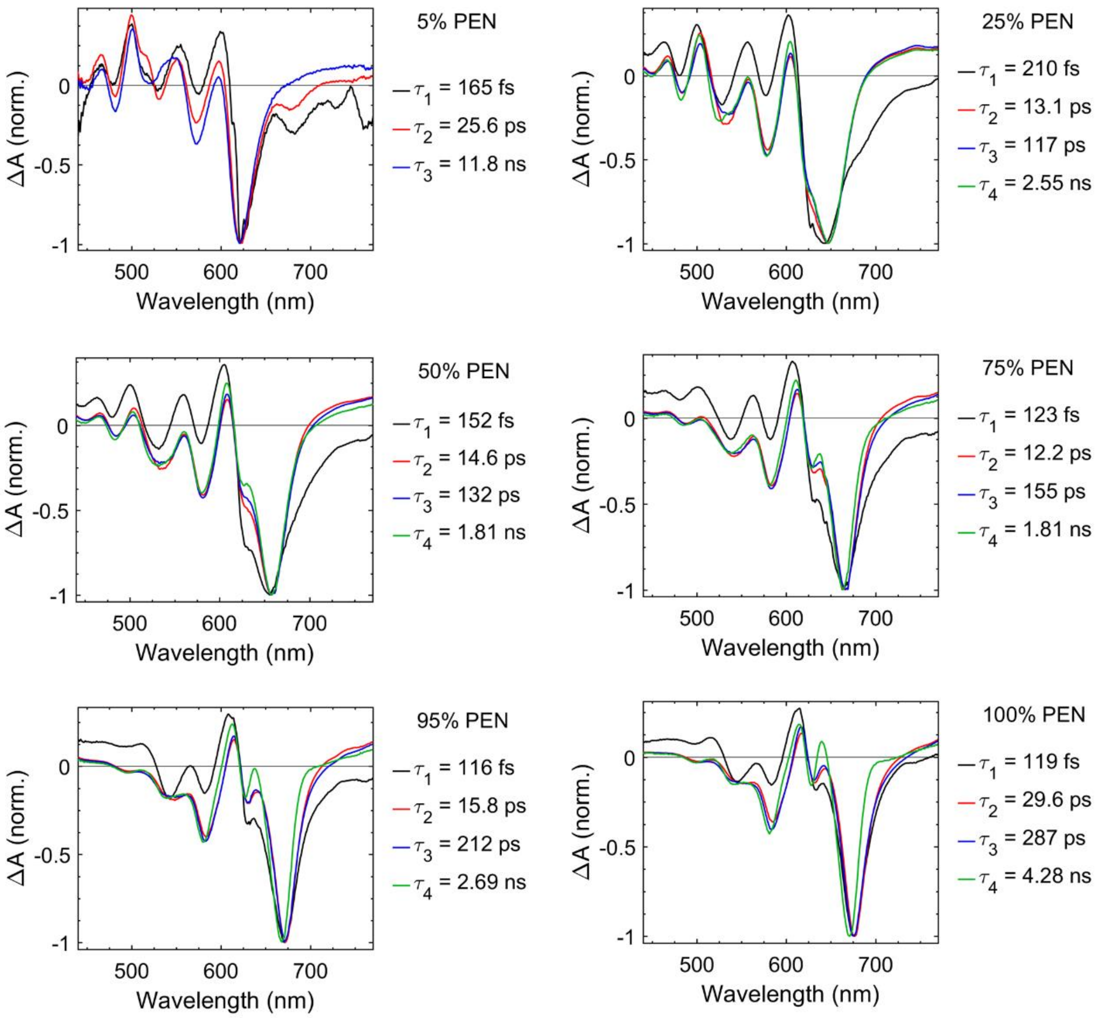

Figure S7: Evolution associated spectra (EAS) and their time constants obtained by GA of the TA measurements at 620 nm excitation wavelength and a pump pulse energy of 70 nJ.

Global analysis (520 nm excitation)

The results of the GA of the TA measurements of PEN:TET blends and neat PEN with 520 nm excitation wavelength are shown in Figure S8. As for the measurements with 620 nm excitation, four EAS are necessary to fit the whole TA map, with three of the EAS being very similar for blends with 25% PEN or more and only being necessary for description of the non-exponential decay of the PEN triplets. We therefore conclude that the photophysics can be described by only two distinct EAS, which now include also TET singlet excitons in addition to the species already described for the measurements with 620 nm excitation wavelength.

For the fit of the TA data of the 5% PEN using an excitation wavelength of 520 nm, four different EAS are necessary in contrast to the measurements using 620 nm as excitation wavelength. The first, second and third EAS mostly differ by the relative intensities of the TET and the PEN GSB. Therefore, the first and the second time constants describe energy transfer from TET to PEN. It is reasonable that this energy transfer exhibits multi-exponential dynamics due to different donor-acceptor distances, which explains the necessity of multiple EAS. We therefore identify the second EAS as an intermediate step of the multi-exponential energy transfer. The third EAS shows a negative peak at 680nm which is most likely stimulated emission from singlet excitons on isolated PEN molecules. These isolated PEN singlets should undergo heterofission within 26 ps as seen in the measurements with 620 nm excitation wavelength. Therefore, even though a complete disentanglement of energy transfer, PEN homofission and heterofission is not possible in the first two EAS, the third time constant of 113 ps mainly reflects the latter, possibly modified by repopulation of the excited singlet state of the isolated PEN molecules by energy transfer. The last EAS corresponds to triplets formed by the two above mentioned processes, since no more stimulated emission is visible. The last time constant consequently describes the decay of these triplets.

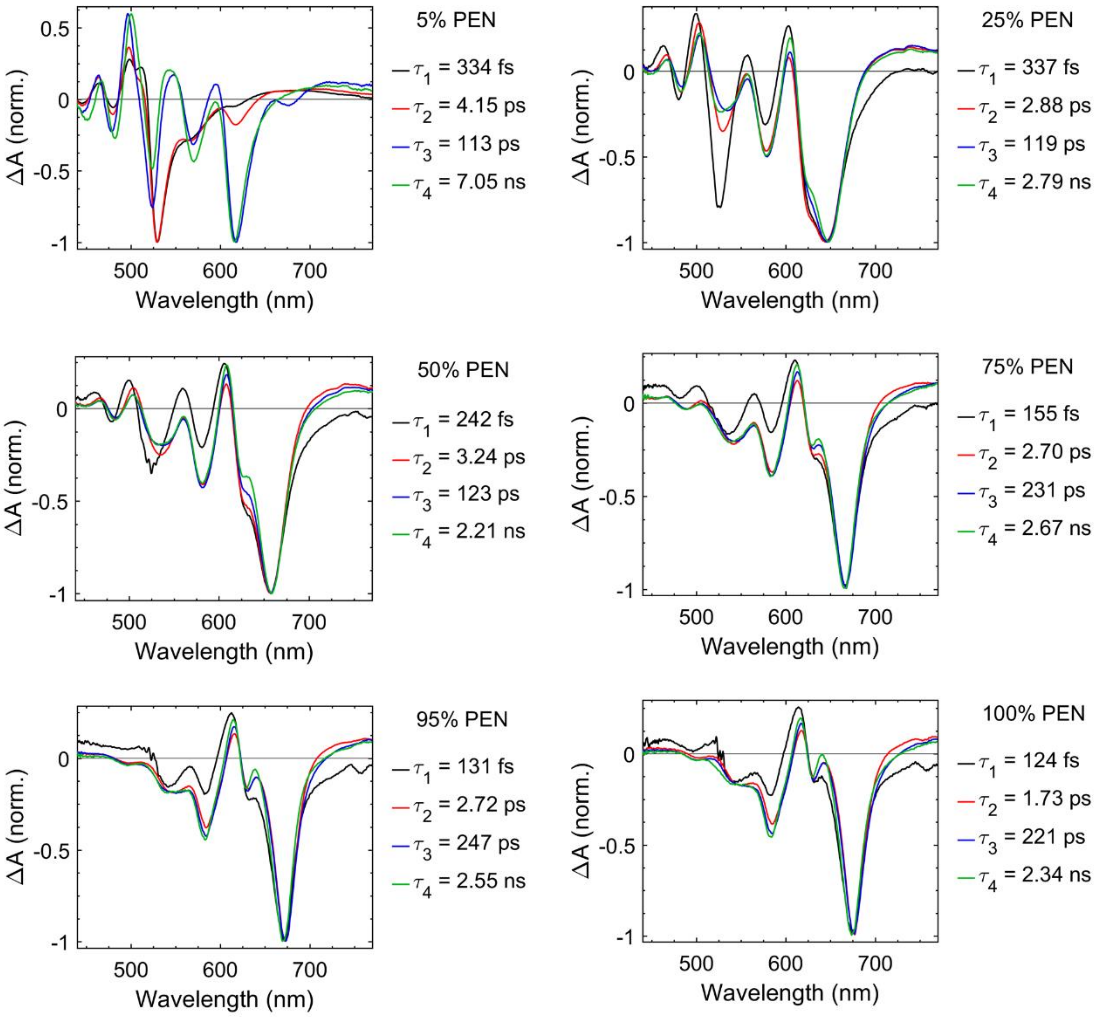

Figure S8: EAS and their time constants obtained by GA of the TA measurements at 520 nm excitation wavelength and a pump pulse energy of 20 nJ.

Comparison of fits and time traces of the 5% PEN blend and fit residuals

Selected time traces of the 5% PEN blend, the corresponding fits resulting from the GA and the fit residuals are shown for 620 nm and 520 nm excitation wavelength in Figure S9 and Figure S10, respectively. The residuals show no systematic deviation and demonstrate the well-defined 26 ps time constant for heterofission.

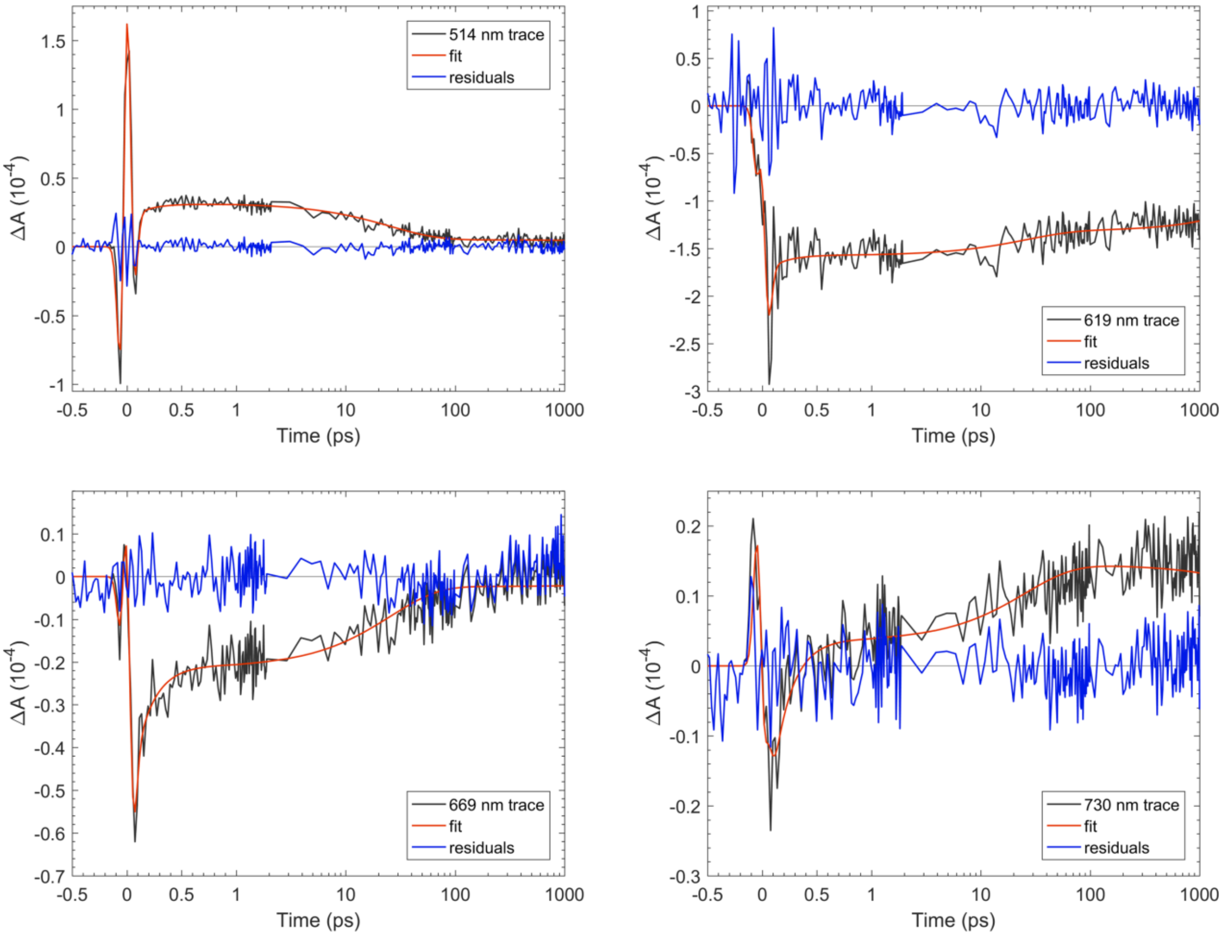

Figure S9: Comparison of time traces and the fits based on GA as well as the fit residuals for selected wavelengths of the 5% PEN blend pumped at 620 nm.

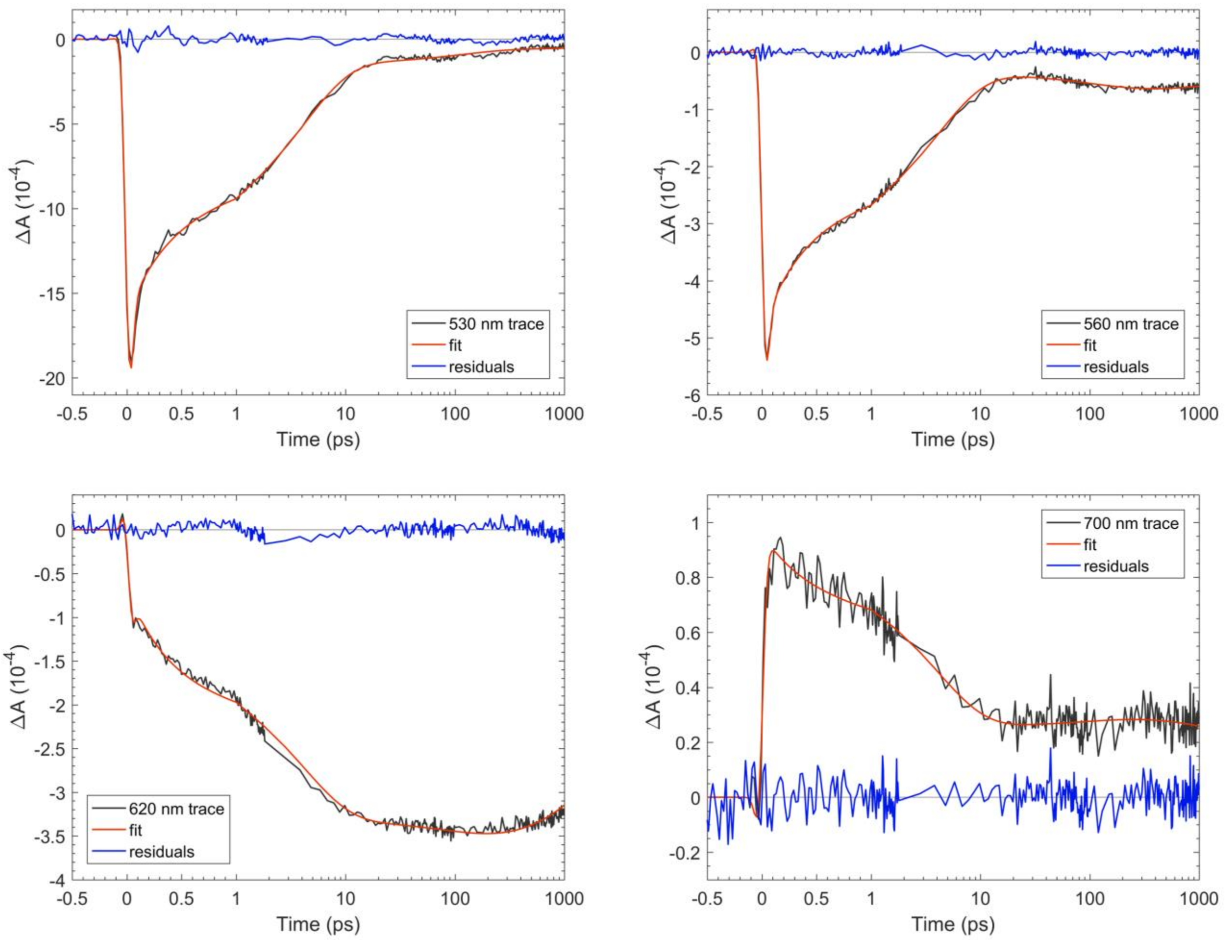

Figure S10: Comparison of time traces and the fits based on GA as well as the fit residuals for selected wavelengths of the 5% PEN blend pumped at 520 nm.

Time trace fits with non-exponential triplet decay

As described before, three of the four EAS obtained by a GA for blends with 25% PEN or more have the same shape and are only needed to describe the non-exponential decay of the triplets formed by PEN SF. This non-exponential dynamics of the triplet decay on a sub-nanosecond timescale in thin films of PEN has been reported before [2-4] and is due to triplet-triplet annihilation (TTA),[3,5] which is a nonlinear (second order) contribution in the rate equation of triplet population. With T(t) being the triplet density at time t and assuming no other decay channels for the triplets, which is reasonable on this timescale, the second order contribution to the rate equation for the triplet density reads [3,4]

$$\frac{dT}{dt} = -k_2 \cdot T(t)^2 \quad ,$$

which has the solution

$$T(t) = \frac{T_0}{T_0 \cdot k_2 \cdot t + 1} \quad ,$$

with the initial triplet density T(0) = T_0. The relative change in the TA signal is therefore

$$\frac{\Delta A(t)}{\Delta A(0)} = \frac{1}{T_0 \cdot k_2 \cdot t + 1} + \delta$$

where we added a fraction δ of triplets which do not participate in the TTA process and decay on a much longer timescale. A fit of this equation to representative time traces using the two parameters $T_0 \cdot k_2$ and δ is shown in Figure S11 along with a multi-exponential fit with three time constants as used for the global analysis. Even though the non-exponential model has less free parameters, it can reasonably well describe the decay of the TA signal in the sub-nanosecond timescale. A more detailed analysis of the triplet decay like the different rate constants k_2 and different δ is beyond the scope of this study.

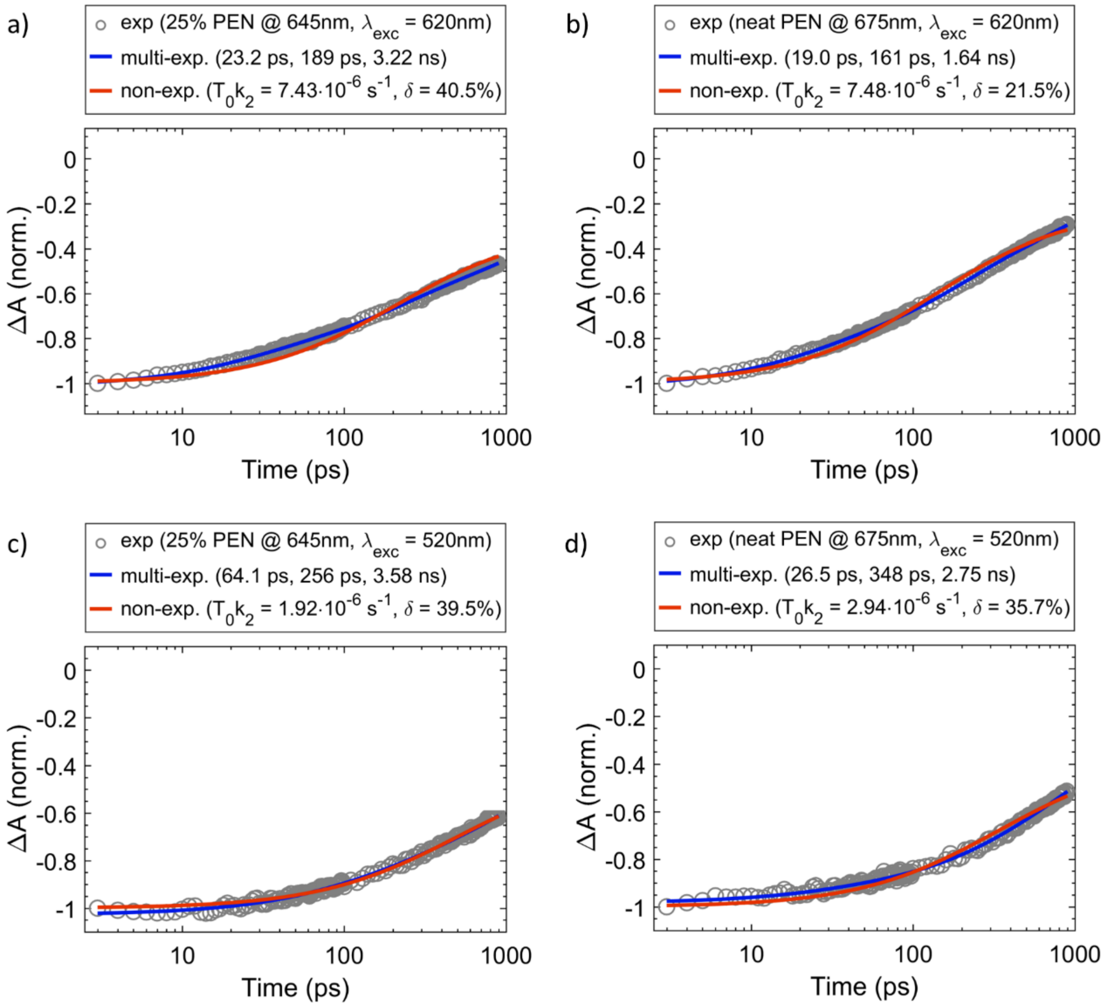

Figure S11: Multi-exponential (blue) and non-exponential (red) triplet decay models for representative measurements (see legend). The wavelengths at which the time traces have been extracted have been chosen to correspond to the PEN GSB.

IR-probe TA measurements (520 nm excitation)

TA spectra in the infrared (IR)-range integrated around 1 ps are shown in Figure S12. Neat PEN triplets produce a strong ESA that is reduced upon dilution of PEN in TET. No new features appear in the blends. Trace dynamics of the TA measurements with IR probe are shown in Figure S13.

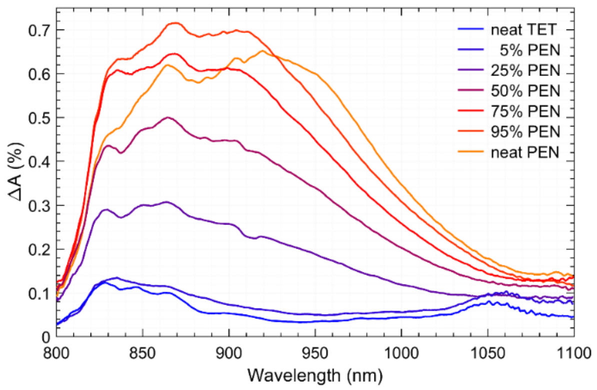

Figure S12: TA spectra of TET:PEN blends at 500-1500 fs with IR probe pulse and 520 nm pump wavelength. The feature at 1050 nm for neat TET and the 5 % blend is most likely the second harmonic of the pump pulse.

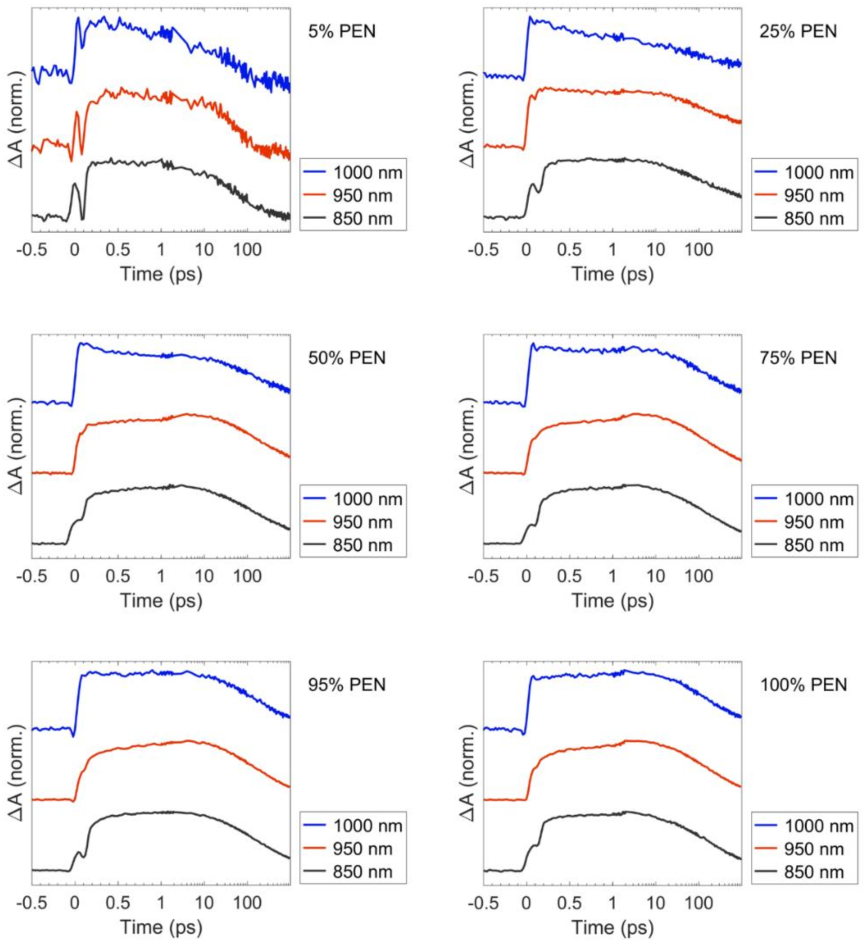

Figure S13: Dynamics at different wavelengths of TA measurements of PEN:TET blends and neat PEN excited at 520 nm and probed in the IR.

Comparison of 25% PEN blends (mixed with TET and PIC) and variable pump energy

In order to confirm the TA features between 450 nm and 520 nm to be thermal artifacts and to exclude a contribution from TET (via two-photon-excitation) or isolated PEN molecules, we performed a fluence-dependence (with λ_{exc} = 620 nm) on the 25% PEN:TET blend. In addition, we performed the same experiments on a PEN:picene (PIC) blend with 25% PEN, having nominally the same probability for isolated PEN molecules.

As seen in Figure S14, the features between 450 nm and 520 nm do not show a fluence-dependence, from which we exclude a contribution of two-photon-excitation of TET. Furthermore, since they are not observed in the PEN:PIC blend (Figure S14b and d), these features also do not originate from isolated PEN molecules. Therefore, we assign these features to thermal artifacts.

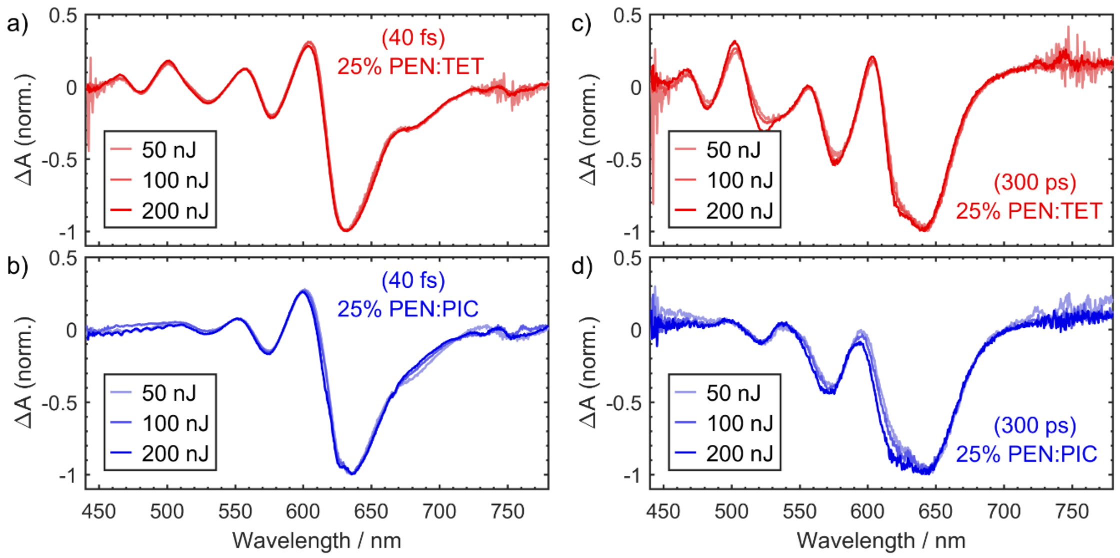

Figure S14: TA spectra at 40 fs and 300 ps of 25 % PEN blends mixed with TET (a,c) and PIC (b,d) at different time delays and pulse energy.

The results of the GA of the TA measurements of a PEN:TET blend with 25% PEN and varying pulse energy are shown in Figure S15. We do not observe a significant dependence of the SF time constant τ_1 on the pump pulse energy.

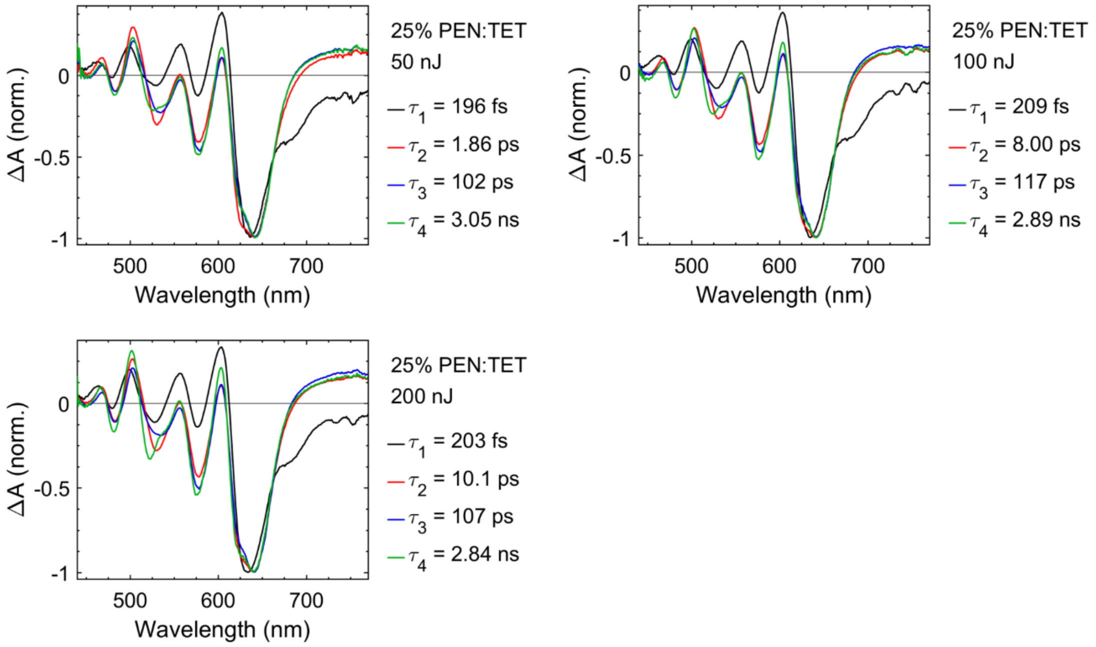

Figure S15: EAS and their time constants obtained by GA of the TA measurements of a PEN:TET blend with 25% PEN and varying pulse energy (see legend).

TA measurements on neat TET

Results from a TA measurement of neat TET are shown in Figure S16. For the GA of this data a three step model was used. Since the first and the second EAS have a similar shape, including features from stimulated emission and singlet absorption above 530 nm, we assign these EASs to singlets and the third one to triplets. The slight shift between the first and the second and third EAS might be due to the decay of the Stokes-shifted SE signal.
It is known that TET singlets decay via singlet-singlet-annihilation (SSA) above a certain exciton density threshold.[5] Since we obtain a lower time constant τ_2 for the decay of the SE (usually representative of the SF process) than those reported before,[5,6] we assume we were above this threshold with the 20 nJ pump pulse energy that we used. We therefore interpret the first time constant τ_1 as an artifact from the non-exponential SSA process, the second time constant as the time constant of TET homofission and the third time constant τ_3 as the decay of triplets via TTA.

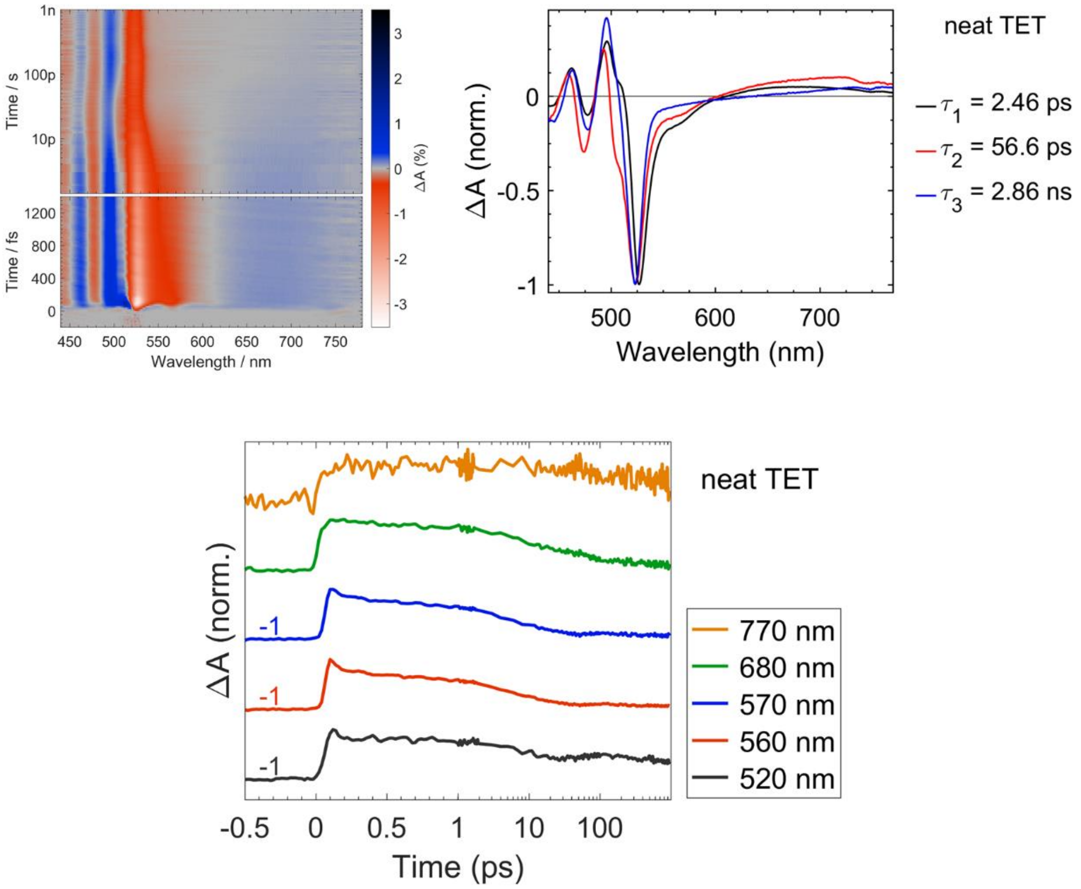

Figure S16: TA map, EAS from GA and time traces of a TA measurement of neat TET with 520 nm excitation wavelength at 20 nJ pump pulse energy. Traces with a negative ΔA signal are shown positive and are labeled with -1.

Comparison of 300 ps spectra of TA measurements with 620 nm and 520 nm excitation

Any contribution of heterofission of a singlet exciton on TET should lead to differences in the TA spectra at long times (300 ps) with high PEN concentrations, where TET homofission is prevented by the isolation of TET molecules. In Figure S17, we compare the TA spectra at 300 ps at the two different pump wavelengths, and find them to almost perfectly agree with each other in blends with 25% PEN concentration or higher. They differ only for the 5% PEN blend, but there is no reason why a TET singlet exciton should undergo heterofission with a neighboring PEN molecule in this blend, but not in the blends with higher PEN concentration and a higher probability of PEN neighbors for a given TET molecule. Although the reason for this difference is not completely clear, it is very unlikely caused by TET heterofission and we exclude the occurence of TET heterofission in all blends.

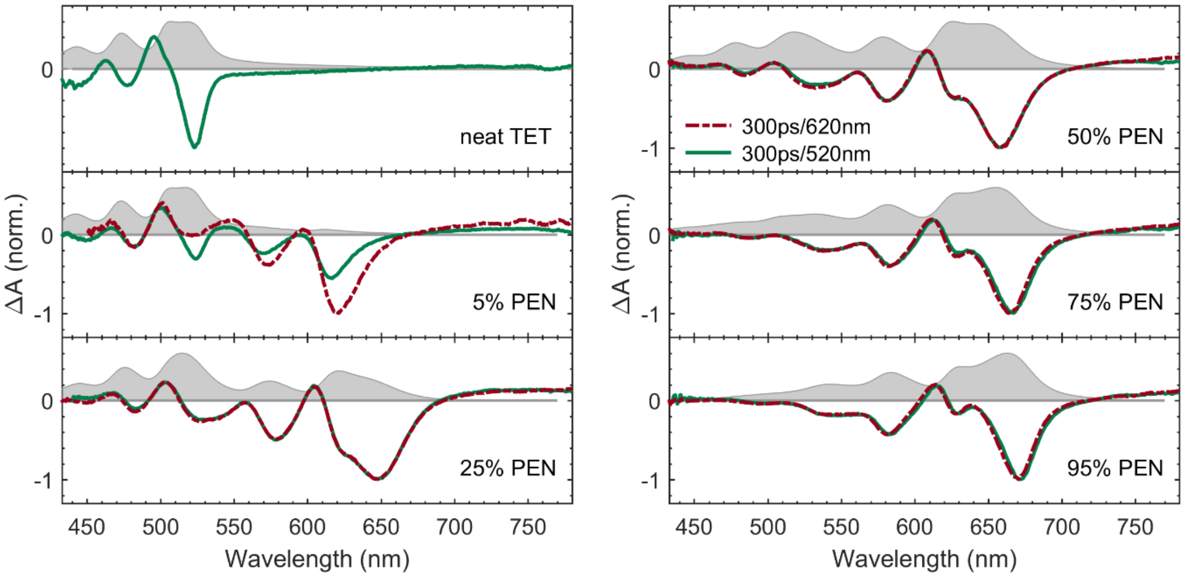

Figure S17: TA spectra of TET:PEN blends at long delays (300 ps) with two different excitation wavelengths. The spectra are normalized to the highest peak except for the 5% PEN data, which are scaled to have the same intensity at 500 nm.

Thermal artifacts

Local heating of the sample caused by the pump pulse is known to lead to thermal artifacts in the TA signal.[7,8] This is caused by a shift of the absorption spectrum of the sample measured after excitation by the pump pulse to higher energies $E+\Delta E$ compared to the absorption spectrum of the sample measured without previous excitation by the pump pulse. [7]
We simulated the contribution of this heating effect to the TA spectra by taking the derivative of the respective absorption spectrum, which is justified since in the calculation of the TA signal the difference between two spectra with and without previous excitation is taken. Therefore, the absorption spectrum thermally shifted after excitation creates a derivative-like contribution to the measured TA signal. [7] Assuming that the shape of the absorption spectrum does not change with temperature and that the energy shift ΔE caused by local heating is small compared to the width of absorption peaks, the thermal artifacts mathematically equal the derivative of the absorption spectrum.

The simulated thermal artifacts are shown for selected blends in Figure S18 and reproduce part of the signals within the region of the PEN and TET GSB and the vibronic progression. Minor deviations might originate from a larger ΔE or a change in the shape of the absorption spectrum. In particular for the 25% PEN:TET blend (Figure S18b), the contribution of thermal artifacts is fully sufficient to describe the spectral features in the wavelength range from 450 nm – 520 nm. This excludes other possible origins of these features, such as the delocalization of the photoexcited bright singlet state over both molecules.[9]
This model for thermal artifacts assumes the heat to be instantaneously distributed homogeneously over the sample independent of the excitation wavelength and therefore is a function solely of the steady-state absorption spectrum. As a noteworthy result, independent of the *excited* compound in a blend, the thermal artifact predominantly occurs at the spectral region of the absorption of the *excess* compound. This leads for 5% PEN:TET even with 620 nm excitation wavelength to a strong thermal artifact in the spectral region of the TET absorption but a negligible thermal artifact in the spectral region of the PEN absorption, see Figure S18e. The amplitude of the thermal artifacts is difficult to determine and is therefore arbitrarily scaled to fit photoinduced absorption features that unambiguously correspond to thermal artifacts.

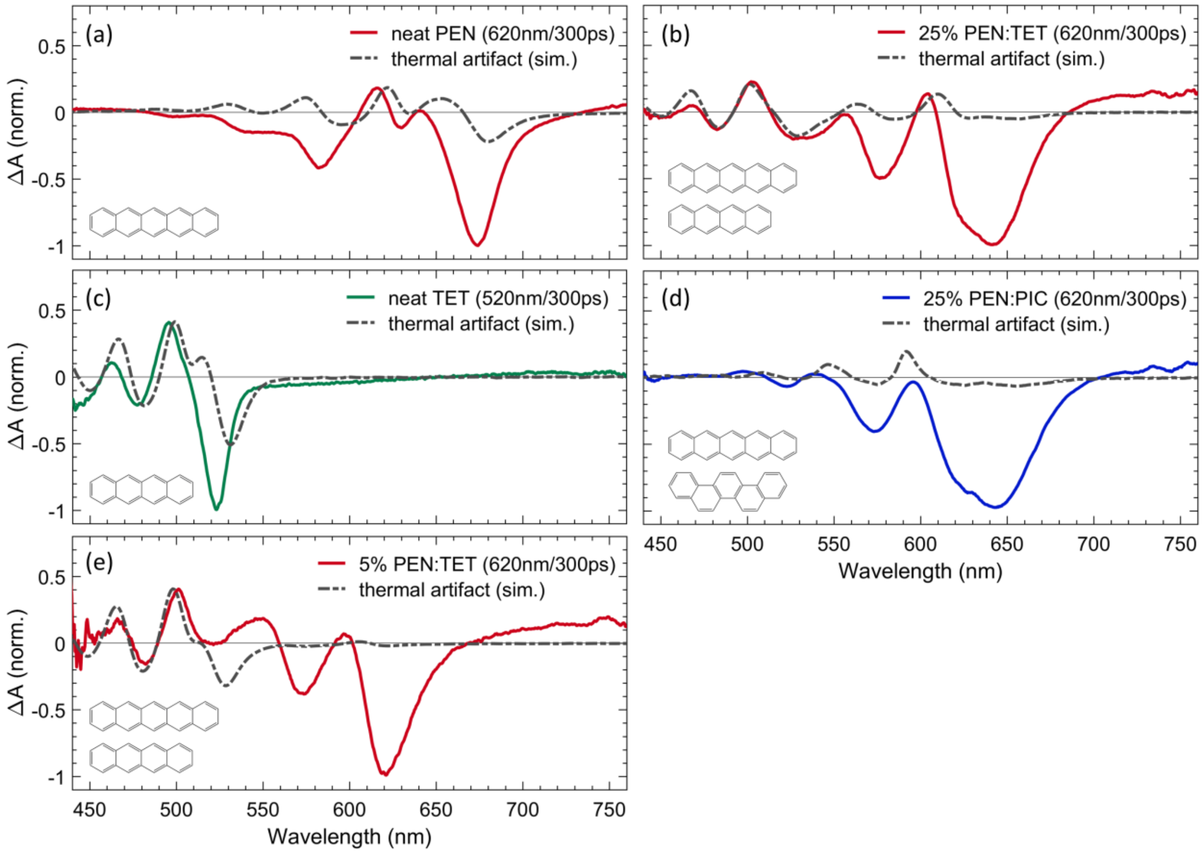

Figure S18: Comparison of the measured TA spectra (solid lines) at long delays (300 ps) with different excitation wavelengths and a simulation of the thermal artifact for: a) PEN, b) 25% PEN:TET blend, c) TET, d) 25% PEN:PIC blend and e) 5% PEN:TET blend, percentages corresponding to the PEN concentration. For details, see text.

Quantiative calculation of spectral contributions in the 5% PEN blend (λ_{exc} = 620 nm)

In order to quantatively estimate the expected contribution of PEN triplets to the TA-spectrum of the 5% PEN blend (λ_{exc} = 620 nm), we scaled the TA-signal of neat PEN at 300 ps, where the signal will be dominated by triplets, with the absorption coefficient of the 5% PEN blend at 620 nm divided by the absorption coefficient of neat PEN at 620nm to account for the reduced number of PEN molecules and the change in spectral shape. The signal has been further divided by 2 to account for the fact that two bleached PEN molecules contribute to the triplet signal after homofission. The results are shown in Figure S19 (red curve). We note that the PEN GSB in the experimental TA-data is blue shifted due to a change in the polarizability of the local molecular environment caused by the TET molecules. This has not been taken into account in the scaling of the simulated TA-signal of neat PEN and explains the difference in the wavelength position of all spectral features (PEN GSB and triplet ESA).
In general, we find good agreement between the simulated (scaled) TA-signal of neat PEN with the absolute values of the experimental TA-data of the 5% PEN blend (black curve) within the region of the PEN GSB. The similar magnitude of the PEN GSB provides evidence for heterofission, which leads to the bleach of one PEN molecule and one TET molecule, whereas homofission would result in two bleached PEN molecules.

We performed the same analysis for TET by scaling the TA-signal of neat TET at 300 ps, where the signal is dominated by triplets, with the absorption coefficient of the 5% PEN blend at 620 nm divided by the absorption coefficient of neat TET at 520 nm, divided by two (see before) and multiplied by 0.8. This was done to account for the fact that in a TA-experiment with an excitation wavelength of 620 nm TET triplets can only be formed by heterofission from PEN monomers (= 80% of all PEN molecules in the 5% blend). Furthermore, the data were scaled to correct for the differences in the pump fluence and photon energy. The result is shown in Figure S19 (blue curve), but any quantitative interpretation is challenging due to the following reasons:
First, since TET does not absorb at 620 nm, the absolute value of the TA-signature of TET triplets in the TA-data of the 5% PEN blend excited with 620 nm is unknown and must be taken from measurements using an excitation wavelength of 520 nm. Since this requires a change in the experimental setup the spot size might differ slightly between the two experiments, which significantly reduces the reliability of a comparison of absolute intensities.
Second, the TA-data of neat TET are affected by a thermal artifact (see Figure S18) and for a determination of absolute values of signals in this wavelength region, which includes the spectral signatures of TET triplets, we have to correct the TA-spectrum of neat TET for this artifact, which is highly challenging in a quantitative way. This further reduces the precision of absolute intensities.
Finally, we do not have clear signatures for TET triplets outside of the region of the TET GSB, but a predicition of absolute values in this wavelength region is complex due to the interference of three signals: (i) The thermal artifact of the 5% PEN blend, (ii) the TET GSB itself and (iii) the PEN singlet ESA.
This makes the prediction of absolute intensities of TET signals highly challenging and we will not attempt an interpretation of the absolute values of the TET spectrum. We note that an analysis of the TA-measurements using an excitation wavelength of 520 nm is not feasible due to energy transfer from TET to PEN.

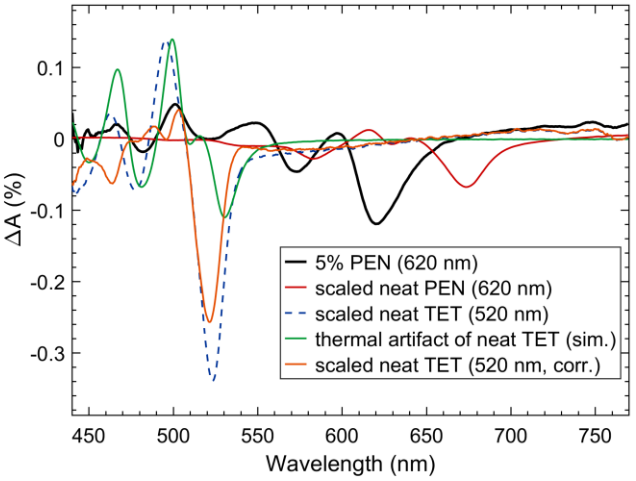

Figure S19: TA spectra of neat PEN (red line) and neat TET (dotted blue line) at 300 ps scaled for comparison of signal amplitudes with the experimental TA data of the 5% PEN blend (black line). For comparison, the thermal artifact of neat TET (green line) and the TA spectra of neat TET with the thermal artifact subtracted (orange line) are shown as well.

Förster radius

The Förster resonance energy transfer (FRET) rate is calculated *via*

$$k_{ET}(R) = k_{rad}\left(\frac{R_F}{R}\right)^6$$

from the radiative rate k_{rad}, the distance R of donor and acceptor and the Förster radius [10-13]

$$R_F^6 = \frac{9\log(10)\,\kappa^2\,J}{2^7\,\pi^5\,n^4\,N_A}$$

Here, J is the overlap integral between the normalized donor emission spectrum and the acceptor molar extinction coefficient [14] which we obtained from the absolute absorbance of a thin film reference with known thickness, κ^2 is the orientation factor between 0 and 4 describing the relative orientation of the transition dipole moments of donor and acceptor,[15,16] n is the refractive index of the medium separating donor and acceptor and N_A is Avogadro's number.

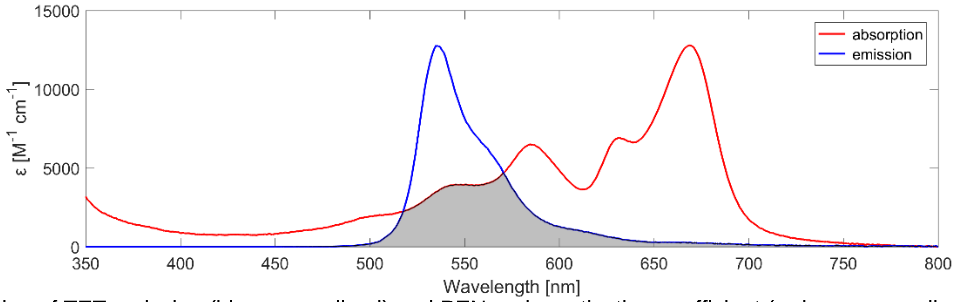

Figure S20: Overlap of TET emission (blue, normalized) and PEN molar extinction coefficient (red, corresponding to left ordinate).[14]

We first calculate the rate of FRET from TET to PEN in vacuum (n=1) with parallel orientation of the transition dipole moment (κ^2=1) and take into account the effects of n≠1 and κ^2≠1 as pre-factors later on. The calculated overlap integral of donor (TET) emission and acceptor (PEN) absorption (see Figure S20) results in a vacuum-Förster-radius of

$$R_F^{vac} = \left[\frac{9\log(10)\,J}{2^7\,\pi^5\,N_A}\right]^{-6} = 5.47\,\text{nm}$$

which is a typical value.[13]

Förster resonance energy transfer rate

Using the Förster radius in vacuum, the total concentration-dependent FRET rate can be calculated by adding the FRET rate of a given donor molecule to a statistical distribution of neighboring acceptor molecules *via*

$$k_{ET}(c) = c\cdot\sum_i k_{ET}(R_i) = c\cdot k_{rad}\cdot n(c)^{-4}\cdot\sum_i \kappa_i^2\left(\frac{R_F^{vac}}{R_i}\right)^6$$

with c the acceptor (PEN) concentration in the film ranging from 0 to 1, $k_{ET}(R_i)$ the FRET rate to a single molecule i, $k_{rad} = 0.0435\,\text{ns}^{-1}$ the radiative decay rate of the donor (TET)[17] n(c) the concentration-dependent refractive index, assumed to be linear between those of TET (n = 1.58 [18]) and PEN (n = 2 [19]), κ_i^2 the orientation-factor for energy transfer to molecule i (Figure S21).[15,16] For the sum over acceptor molecules we consider 24 neighbors of a given donor molecule (0 in Figure S21) in a herringbone lattice. For each neighbor, the orientation factors κ_i^2 and distances R_i can be calculated for a TET bulk[20] and a PEN thin film[21] structure (see tables below). By far, the fastest FRET occurs between translationally equivalent molecules along the short axis of the herringbone lattice. Since for both compounds and structure types (bulk / thin film) most of the κ_i^2 are similar, we used those of thin film PEN to calculate the FRET rate.

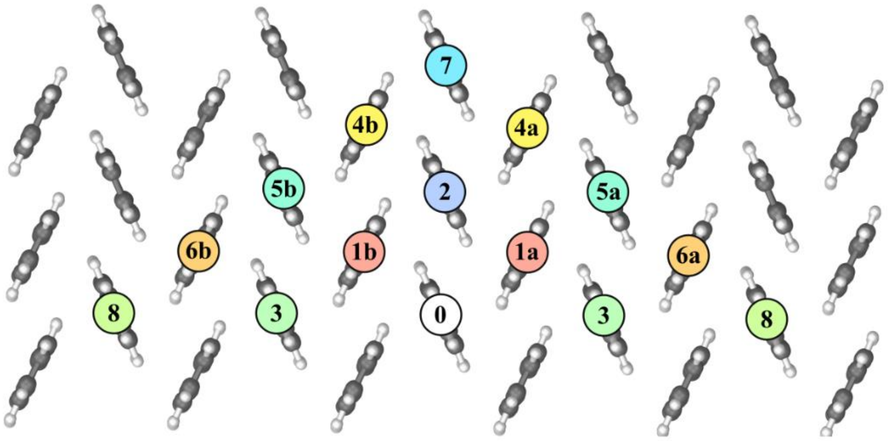

Pentacene

target	κ^2	d [Å]	κ^2 d^{-6} [$Å^{-6}$]	relative
1a	0.006	4.819	4.791 E-07	1.00
1b	0.005	4.835	3.914 E-07	0.82
2	1.838	5.958	4.109 E-05	85.77
2'	1.817	5.958	4.062 E-05	84.79
3	0.146	7.596	7.601 E-07	1.59
3'	0.136	7.596	7.080 E-07	1.48
4a	1.725	9.723	2.042 E-06	4.26
4b	1.724	9.698	2.072 E-06	4.33
5a	0.796	9.67	9.735 E-07	2.03
5a'	2.127	9.67	2.601 E-06	5.43
5b	2.133	9.637	2.663 E-06	5.56
5b'	0.804	9.637	1.004 E-06	2.10
6a	1.027	11.787	3.830 E-07	0.80
6b	1.044	11.767	3.933 E-07	0.82
7	1.838	11.916	6.420 E-07	1.34
7'	1.817	11.916	6.347 E-07	1.32
8	0.146	15.192	1.188 E-08	0.02
8'	0.136	15.192	1.106 E-08	0.02

Tetracene

target	κ^2	d [Å]	κ^2 d^{-6} [$Å^{-6}$]	relative
1a	0.013	5.125	7.174 E-07	1.50
1b	0.004	4.773	3.383 E-07	0.71
2	1.704	6.057	3.451 E-05	72.03
2'	1.49	6.057	3.017 E-05	62.98
3	0.252	7.838	1.087 E-06	2.27
3'	0.248	7.838	1.070 E-06	2.23
4a	1.261	10.155	1.150 E-06	2.40
4b	1.693	9.626	2.128 E-06	4.44
5a	0.761	10.251	6.558 E-07	1.37
5a'	1.047	10.251	9.023 E-07	1.88
5b	1.903	9.547	2.513 E-06	5.25
5b'	0.753	9.547	9.945 E-07	2.08
6a	0.873	12.353	2.457 E-07	0.51
6b	0.94	11.923	3.272 E-07	0.68
7	1.704	12.113	5.395 E-07	1.13
7'	1.49	12.113	4.717 E-07	0.98
8	0.252	15.675	1.699 E-08	0.04
8'	0.248	15.675	1.672 E-08	0.03

Figure S21: Typical herringbone lattice with numbers for different FRET targets starting from a donor molecule 0. The targets can be point-mirrored to the lower half. The tables show calculated orientation factors and distances for a TET bulk and a PEN thin film structure.[20,21] The targets with a prime denote donor-target combinations, for which the orientation factor differs if the donor/target assignment is displaced to a molecule translationally inequivalent to molecule 0 (e.g. 1a).

References

[1] J. J. Snellenburg, S. Laptenok, R. Seger, K. M. Mullen, I. H. M. Van Stokkum, *J. Stat. Softw.* **2012**, *49*.
[2] H. Marciniak, M. Fiebig, M. Huth, S. Schiefer, B. Nickel, F. Selmaier, S. Lochbrunner, *Phys. Rev. Lett.* **2007**, *99*, 176402.
[3] A. D. Poletayev, J. Clark, M. W. B. Wilson, A. Rao, Y. Makino, S. Hotta, R. H. Friend, *Adv. Mater.* **2013**, *26*, 919–924.
[4] C. Grieco, G. S. Doucette, R. D. Pensack, M. M. Payne, A. Rimshaw, G. D. Scholes, J. E. Anthony, J. B. Asbury, *J. Am. Chem. Soc.* **2016**, *138*, 16069–16080.
[5] J. J. Burdett, D. Gosztola, C. J. Bardeen, *J. Chem. Phys.* **2011**, *135*, 214508.
[6] M. W. B. Wilson, A. Rao, K. Johnson, S. Gélinas, R. di Pietro, J. Clark, R. H. Friend, *J. Am. Chem. Soc.* **2013**, *135*, 16680–16688.
[7] A. Rao, M. W. B. Wilson, S. Albert-Seifried, R. Di Pietro, R. H. Friend, *Phys. Rev. B* **2011**, *84*, 195411.
[8] M. W. B. Wilson, A. Rao, J. Clark, R. S. S. Kumar, D. Brida, G. Cerullo, R. H. Friend, *J. Am. Chem. Soc.* **2011**, *133*, 11830–11833.
[9] S. N. Sanders, E. Kumarasamy, A. B. Pun, M. L. Steigerwald, M. Y. Sfeir, L. M. Campos, *Angew. Chem.* **2016**, *128*, 3434–3438.
[10] A. Köhler, H. Bässler, *Electronic Processes in Organic Semiconductors: An Introduction*, John Wiley & Sons, **2015**.
[11] T. Förster, *Radiat. Res. Suppl.* **1960**, 326–339.
[12] G. Cerullo, S. Stagira, M. Zavelani-Rossi, S. De Silvestri, T. Virgili, D. G. Lidzey, D. D. C. Bradley, *Chem. Phys. Lett.* **2001**, *335*, 27–33.
[13] M. Mitsui, Y. Kawano, *Chem. Phys.* **2013**, *419*, 30–36.
[14] L. B.-Å. Johansson, F. Bergström, P. Edman, I. V. Grechishnikova, J. G. Molotkovsky, *J. Chem. Soc., Faraday Trans.* **1996**, *92*, 1563–1567.
[15] L. Loura, *Int. J. Mol. Sci.* **2012**, *13*, 15252–15270.
[16] A. Burghart, L. H. Thoresen, J. Chen, K. Burgess, F. Bergström, L. B.-Å. Johansson, *Chem. Commun.* **2000**, 2203–2204.
[17] A. M. Müller, Y. S. Avlasevich, W. W. Schoeller, K. Müllen, C. J. Bardeen, *J. Am. Chem. Soc.* **2007**, *129*, 14240–14250.
[18] B. Gompf, D. Faltermeier, C. Redling, M. Dressel, J. Pflaum, *Eur. Phys. J. E* **2008**, *27*, 421–424.
[19] M. Dressel, B. Gompf, D. Faltermeier, A. K. Tripathi, J. Pflaum, M. Schubert, *Opt. Express* **2008**, *16*, 19770–19778.

[20] D. Holmes, S. Kumaraswamy, A. J. Matzger, K. P. C. Vollhardt, *Chem. Eur. J.* **1999**, *5*, 3399–3412.
[21] S. Schiefer, M. Huth, A. Dobrinevski, B. Nickel, *J. Am. Chem. Soc.* **2007**, *129*, 10316–10317.
